# Three-Dimensional-Printed Vortex Tube Reactor for Continuous Flow Synthesis of Polyglycolic Acid Nanoparticles with High Productivity

**DOI:** 10.3390/nano13192679

**Published:** 2023-09-29

**Authors:** Kittipat Suwanpitak, Pornsak Sriamornsak, Inderbir Singh, Tanikan Sangnim, Kampanart Huanbutta

**Affiliations:** 1 Faculty of Pharmaceutical Sciences, Burapha University, Chonburi 20131, Thailand; kittipatsuwanpitak@gmail.com; 2 Department of Industrial Pharmacy, Faculty of Pharmacy, Silpakorn University, Nakhon Pathom 73000, Thailand; sriamornsak_p@su.ac.th; 3 Academy of Science, The Royal Society of Thailand, Bangkok 10300, Thailand; 4 Faculty of Pharmaceutical Sciences, Chulalongkorn University, Bangkok 10330, Thailand; 5 Chitkara College of Pharmacy, Chitkara University, Patiala 140401, Punjab, India; inderbir.singh@chitkara.edu.in; 6 Department of Manufacturing Pharmacy, College of Pharmacy, Rangsit University, Pathum Thani 12000, Thailand

**Keywords:** flow chemistry, computational fluid dynamics, 3D printing, PGA nanoparticles

## Abstract

Polyglycolic acid (PGA) nanoparticles show promise in biomedical applications due to their exceptional biocompatibility and biodegradability. These nanoparticles can be readily modified, facilitating targeted drug delivery and promoting specific interactions with diseased tissues or cells, including imaging agents and theranostic approaches. Their potential to advance precision medicine and personalized treatments is evident. However, conventional methods such as emulsification solvent evaporation via batch synthesis or tubular reactors via flow chemistry have limitations in terms of nanoparticle properties, productivity, and scalability. To overcome these limitations, this study focuses on the design and development of a 3D-printed vortex tube reactor for the continuous synthesis of PGA nanoparticles using flow chemistry. Computer-aided design (CAD) and the design of experiments (DoE) optimize the reactor design, and computational fluid dynamics simulations (CFD) evaluate the mixing index (MI) and Reynolds (Re) expression. The optimized reactor design was fabricated using fused deposition modeling (FDM) with polypropylene (PP) as the polymer. Dispersion experiments validate the optimization process and investigate the impact of input flow parameters. PGA nanoparticles were synthesized and characterized for size and polydispersity index (PDI). The results demonstrate the feasibility of using a 3D-printed vortex tube reactor for the continuous synthesis of PGA nanoparticles through flow chemistry and highlight the importance of reactor design in nanoparticle production. The CFD results of the optimized reactor design showed homogeneous mixing across a wide range of flow rates with increasing Reynolds expression. The residence time distribution (RTD) results confirmed that increasing the flow rate in the 3D-printed vortex tube reactor system reduced the dispersion variance in the tracer. Both experiments demonstrated improved mixing efficiency and productivity compared to traditional tubular reactors. The study also revealed that the total flow rate had a significant impact on the size and polydispersity index of the formulated PGA nanoparticle, with the optimal total flow rate at 104.46 mL/min, leading to smaller nanoparticles and a lower polydispersity index. Additionally, increasing the aqueous-to-organic volumetric ratio had a significant effect on the reduced particle size of the PGA nanoparticles. Overall, this study provides insights into the use of 3D-printed vortex tube reactors for the continuous synthesis of PGA nanoparticles and underscores the importance of reactor design and flow parameters in PGA nanoparticle formulation.

## 1. Introduction

Polyglycolic acid (PGA) nanoparticles have gained significant attention in recent years as promising nanocarriers for various biomedical applications. PGA, a biocompatible and biodegradable polymer, possesses unique properties such as high water solubility, stability, and a high payload capacity, making it an ideal candidate for drug delivery systems. The small size of PGA nanoparticles, typically in the range of tens to hundreds of nanometers, allows for efficient cellular uptake and enhanced bioavailability. Moreover, their surface can be easily modified for targeted drug delivery, enabling specific interactions with diseased tissues or cells. With their favorable physicochemical properties and versatile surface functionalization, PGA nanoparticles hold great potential for revolutionizing drug delivery, imaging agents, and theranostic applications, contributing to advancements in precision medicine and personalized treatments. The formulation of PGA nanoparticles is typically carried out using emulsification solvent evaporation via batch synthesis methods, which involve the mixing of the organic phase, consisting of PGA, and the aqueous phase, followed by the evaporation of organic solvent [1]. However, batch synthesis methods, which are the regular method to prepare PGA nanoparticles, are associated with several limitations, including low productivity and poor scalability [2]. These limitations have led to increasing interest in the development of continuous synthesis methods, such as flow chemistry, for the production of PGA nanoparticles.

Flow chemistry, also known as continuous flow chemistry or continuous flow synthesis, involves conducting chemical reactions in a continuous flow of reagents through a reactor. The adoption of flow chemistry has revolutionized pharmaceutical manufacturing, enabling the rapid synthesis of active pharmaceutical ingredients (APIs), pharmaceutical excipient, and formulation, including nanoparticle synthesis. Flow chemistry has emerged as a promising approach for nanoparticle synthesis, offering several distinct advantages. Through precise control over reaction parameters, such as temperature, pressure, and residence time, flow chemistry allows one to achieve better control over the size, shape, and composition of nanoparticles, ensuring reproducibility and reducing batch-to-batch variations. Additionally, the rapid mixing and efficient heat transfer in flow systems improve reaction kinetics, resulting in nanoparticles with narrow size distributions, high purity, and improved crystallinity. Flow chemistry also offers scalability and high throughput, allowing for large-scale production and the rapid screening of reaction conditions. Moreover, the safety and sustainability aspects of flow chemistry, with reduced risk in handling hazardous materials and minimized formation of undesired by-products, further enhance its appeal as a technique for nanoparticle synthesis [3,4,5,6,7].

However, the utilization of general reactors, such as tubular reactors or coil reactors, in nanoparticle synthesis though flow chemistry presents several noteworthy limitations that necessitate careful consideration. One such limitation is the challenge of scaling up the synthesis process. As the reactor volume increases, maintaining uniform flow conditions and consistent heat and mass transfer becomes more complex, hindering the large-scale production of nanoparticles. This limitation is closely linked to the design of the tubular reactor. Factors such as reactor diameter, length, and configuration directly impact flow characteristics and residence time distribution, ultimately affecting the mixing efficiency. Furthermore, the flow regime inside the reactor significantly influences the overall mixing efficiency. Laminar flow, characterized by smooth, parallel layers of fluid, generally results in poor mixing, while turbulent flow, with its chaotic fluid motion, promotes better mixing by increasing the contact between reactants. The flow characteristics being examined are dependent on the flow rate utilized in the synthesis process. It is important to note that tubular reactors have a limited range of flow rates in which optimal mixing efficiency can be achieved [8,9]. Nevertheless, ongoing research and technological advancements are focused on overcoming these limitations and new reactor designs are needed to address this issue, aiming to enhance the effectiveness and broaden the applicability of flow chemistry reactors. The design of a reactor’s geometry plays a crucial role in determining flow streamlines and, subsequently, mixing behavior. When streamlines are parallel or minimally interact, there is limited mixing between fluid streams, observed in laminar flows. Conversely, convoluted, intersecting, and complex streamline patterns enhance mixing, characteristic of turbulent flows. Turbulence facilitates the exchange of momentum, energy, and mass, promoting effective mixing through flow instabilities, eddies, and vortices. These structures displace and intermingle fluid particles, resulting in a more homogeneous distribution of properties. Accordingly, mixing efficiency is achieved by promoting vortex patterns in the reactor’s design [10,11].

The present study focuses on the design and development of a 3D-printed vortex tube reactor to improve the productivity of PGA nanoparticles via flow chemistry. The optimization of the reactor design was achieved through the use of computer-aided design (CAD) and the design of experiments (DOE). CAD software was employed to model the reactor geometry, while DOE methodology, central composite design, was used to systematically study the effect of different design parameters on the performance of the reactor. In the evaluation of the reactor models, computational fluid dynamics (CFD) simulations were utilized to evaluate the mixing performance and the flow characteristics within the reactor. Mixing efficiency is a critical factor in the synthesis process as it affects both the rate and uniformity of the reaction. To quantify the degree of homogeneity in the mixing process, a mixing index was employed as a quantitative measure [12]. Additionally, the flow characteristics of the reactors were evaluated. One key parameter used to analyze the flow characteristics is the Reynolds number, which quantifies the relative importance of inertial forces to viscous forces in the fluid flow. By calculating the Reynolds number, we were able to assess the flow regime within the reactor and determine whether it was laminar or turbulent [13,14]. Incorporating CFD simulations with DoE methodology allowed for a comprehensive evaluation of both the mixing performance and flow characteristics, facilitating the refinement and enhancement of the optimized vortex tube reactor model.

Furthermore, the optimized reactor design was fabricated using fused deposition modeling (FDM), a popular 3D printing technique. The principle of FDM involves melting and extruding thermoplastic materials through a nozzle, layer by layer, to create a three-dimensional object from CAD model. The choice of polypropylene (PP) as the polymer for the fabrication of the vortex tube reactor was based on its favorable properties, including high temperature resistance, high strength, and good chemical resistance [15]. In assessment of the 3D-printed vortex tube reactor, the dispersion experiments were conducted to study the residence time distribution (RTD) and deviation within the reactor. RTD is a measure of the time that a fluid particle spends within the reactor, and deviation is a measure of the uniformity of the dispersion. These experiments provided crucial information on the performance of the reactor and helped to validate the optimization process [16]. Finally, the results of this study hold significant implications for the effect of flow parameters, which consist of the total flow rate and the aqueous-to-organic volumetric ratio, in the formulation of PGA nanoparticle production through flow chemistry. The study demonstrates the feasibility of using a 3D-printed vortex tube reactor for this purpose [17]. By utilizing the 3D-printed vortex tube reactor, the researchers achieved efficient control over the flow parameters, enabling precise manipulation of the total flow rate and aqueous-to-organic volumetric ratio during PGA nanoparticle production. This novel approach not only paves the way for the scalable and reproducible synthesis of PGA nanoparticles but also opens up new avenues for further research in this area, such as exploring the impact of different flow parameter combinations on nanoparticle characteristics and optimizing the reactor design for enhanced performance.

## 2. Materials and Methods

### 2.1. Materials

Polyglycolic acid (PGA), cosmetic grade, was purchased from Chemipan Corporation Co., Ltd. (Bangkok, Thailand). Polyvinyl alcohol (PVA), laboratory grade, was purchased from Sigma Aldrich, Inc. (St. Louis, MO, USA). Erythrosine, food grade, was purchased from Adinop Co., Ltd. (Bangkok, Thailand). Dichloromethane, laboratory grade, was purchased from RCI Labscan Ltd. (Bangkok, Thailand). Acetone, laboratory grade, was purchased from QRëC (Chonburi, Thailand). Polypropylene (PP) filament was obtained from B and Brothers Co., Ltd. (Samut Sakhon, Thailand).

### 2.2. The Vortex Tube Reactor Model Design and Model Development according to Design of Experiment

**Figure 1:**
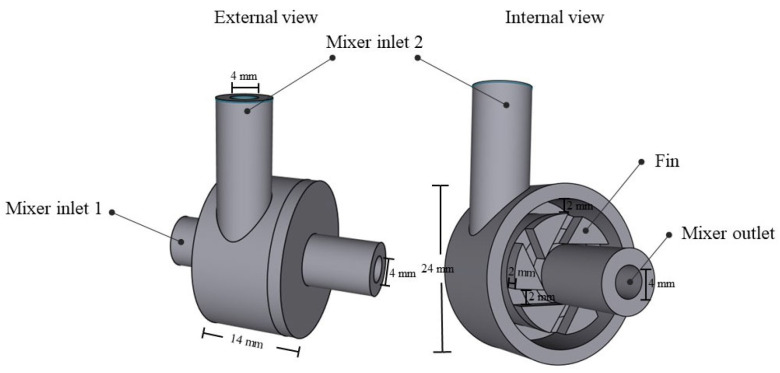
The designed mixer model.

The design of the vortex tube reactor was carried out using Shapr3D software (version number: 5.450.0.5689 #a0a93e9a (Educational license)). The reactor designed for flow chemistry is composed of two main components, which are a mixer and a vortex tube reactor. The mixer part design of the reactor is shown in Figure 1. The model is composed of two inlets (4 mm diameter), and the flow streamlines at mixer inlet 2 are interconnected through a fin for mixing with the flow streamline at mixer inlet 1. The fluid flow is moved tangentially within the mixer part, and the whirling fluid flow is then forced by the fin to change direction and form a vortex streamline before exiting the mixer through the outlet. This vortex streamline then passes through the reactor.

**Figure 2:**
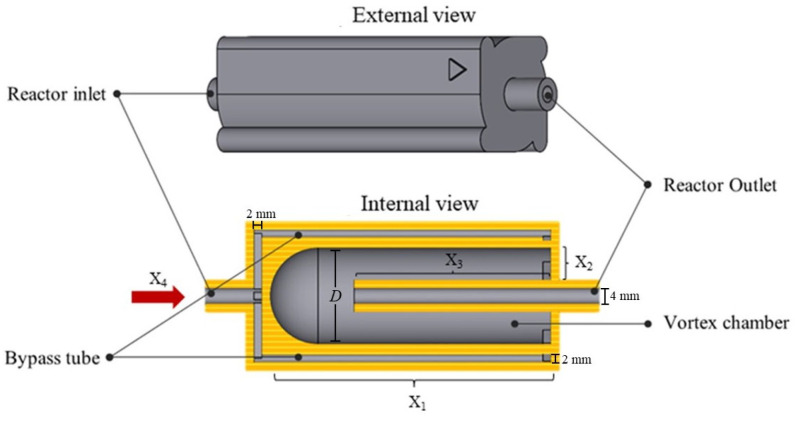
The designed vortex tube reactor model.

The vortex tube reactor in this flow chemistry system is shown in Figure 2. The model is designed with a focus on mixing performance and flow characteristics. The reactor is designed to create a vortex streamline that enhances mixing and mass transfer, resulting in greater productivity of the streamlines which are separated into 4 parts of the by-pass tube, intended to increase fluid velocity, which creates a suitable environment for vortex mixing of the reagents inside the chamber.

The design of the reactor and the operating parameters were studied and optimized using the experimental design (DoE), the central composite design. As presented in Table 1, the interest factors in this study were reactor length (X_1_), internal tube to chamber wall width (X_2_), percentage of internal tube length per reactor length (X_3_), and total flow rate (X_4_). Moreover, the experimental runs were generated and tested using CFD (Flow simulation, Solidworks2021), and then the following responses, including mixing index (Y_1_) and Reynolds number (Y_2_), were monitored and recorded.

### 2.3. Numerical Analysis

The mathematical models and equations were used to conduct the computational fluid dynamics (CFD) simulations. The simulations were run using flow simulation software (Solidworks2021). These equations take into account the effects of viscosity, pressure, and density to predict the flow behavior in a particular system. The governing equations of the three-dimensional, steady, and incompressible flows are the Navier–Stokes equations which can be solved using the finite element method (FEM) or finite volume method (FVM). The Navier–Stokes equations are given in the following:

(1) Continuity equation:  ∇·V=2

(2) $$\mathrm{Momentum}\;\mathrm{equation}:\;\rho \left(\frac{\partial V}{\partial t}+(V\cdot \nabla )V)\right)\;=-\nabla P+\mu \nabla^{2}V$$

where *C* is the species concentration, *D* is the diffusion coefficient, and *V* is the fluid velocity.

The species transport model was employed as it is the most commonly used method to model the mixing of miscible fluids, taking into account the mass diffusion coefficient. The code solved the conservation equations that described the sources of convection and diffusion for each species. The local mass fraction of each species was predicted by solving the convection–diffusion equation, with the sum of the mass fractions of all species being equal to one.

The CFD code was used to solve the governing equations of three-dimensional, steady, and incompressible flows (continuity and momentum). The species transport model, which takes into account the mass diffusion coefficient, was also used. The conservation equations for each species were solved by considering convection and diffusion mechanisms. The liquid properties from the engineering database (Solidworks), shown in Table S1 in the Supplementary Materials, were used for CFD calculation, and the mixing was governed mainly by chaotic advection. The boundary conditions were rigid and non-movable walls, a non-slip velocity condition, and constant uniform velocity at the inlets. The mass fraction ratio of water/ethanol was equal to 1:0 at inlet 1 and 0:1 at inlet 2, while at the outlet, environment pressure and temperature at 25 °C were assumed.

#### 2.3.1. Mixing Index Evaluation

The evaluation of a mixing index (MI) is established based on the deviation of the mass fraction in a cross-section. This method involves dividing the cross-section into several cells, which in this case are the grid elements. The mass fraction is then calculated for each cell. Ideal mixing is characterized by a uniform distribution of particles from the two branches throughout the cross-section, resulting in equal proportions of particles with indices 0 and 1. Optimal mixing should therefore result in an even partition of particles with indices 0 and 1. The calculation of the MI in each cross-section of the micromixer is conducted as follows:

(3) $$MI=1-\frac{\sigma }{\sigma_{0}}$$

where σ is the standard deviation of the mass fraction in a cross-section, which is determined using the integrated function of the CFD code:

(4) $$\sigma^{2}=\frac{1}{N}\sum_{i=1}^{N}{\left(C_{i}-\bar{C}\right)}^{2}$$

The standard deviation is at its maximum for unmixed fluids and at its minimum for perfectly mixed fluids. *N* represents the total number of cells in a cross-section, and $\bar{C}$ is the average mass fraction. Mixing is considered to have occurred when the mass fractions of the two fluids are equal, meaning that they both reach the value of 0.5. The maximum standard deviation σ_0_ over the data range is calculated as follows:

(5) $$\sigma_{0}^{2}=\bar{C}(1-\bar{C})$$

A mixing index of MI = 1 indicates perfect mixing (σ = 0), and MI = 0 indicates an unmixed state (σ = σ_0_). A higher value of MI indicates a more homogeneous concentration and better mixing performance [12].

#### 2.3.2. Reynolds Number Expression

The generalized Reynolds number (Re) expression for the vortex tube reactor was calculated using computational fluid dynamics (CFD) simulations, seen in the following equation:

(6) $$Re=\frac{\rho vD}{\mu }$$

The following parameters were used in the calculation of the Reynolds number expression. Fluid density (*ρ*) and dynamic viscosity (*μ*) were taken as inputs based on the fluid used in the vortex tube reactor, which was obtained from an engineering database (Solidworks2021) that provides the values for the fluid as a function of temperature, as seen in Table S1 in the Supplementary Materials. The velocity is characterized by the average fluid inlet velocity (*v*) that is calculated using the integrated function of the CFD code. The velocity was determined by measuring the fluid flow rate and dividing it by the cross-sectional area of the reactor, and the entire fluid flow was assumed to exit through the fluid outlet of the vortex tube reactor. The diameter (*D*) of the reactor was taken as an input, based on the overall body of the vortex tube reactor [13,18,19].

### 2.4. Mesh Independency Test

This sub-section presents a detailed methodology for the mesh sensitivity analysis conducted in the vortex tube reactor study. To evaluate the sensitivity of the simulation results, comprehensive mesh sensitivity analysis was conducted for each mesh resolution. The analysis involved systematically varying seven levels of automated meshing while keeping the other simulation parameters constant. There are two types of mesh: the tetrahedral shape is suitable for curved surfaces, and the hexahedral shape is appropriate for flat surfaces. The difference in mesh refinement is shown in Table S2 in the Supplementary Materials. By comparing the simulation results obtained from different mesh resolutions, the convergence and stability of the results were assessed. The analysis aimed to determine the optimal mesh size and resolution that would ensure accurate and reliable results in the numerical simulations.

### 2.5. OptiMization of Geometrical Vortex Tube Reactor Model Design

Once the simulation of each sample is completed, the Design-Expert 11 software is used to calculate the relationship patterns using partial least square models and 3D surface plots. Then, the optimized vortex tube reactor is calculated using the setting criteria shown in Table 2, where Y_1_ means that the mixing index must be close to 1 (target to 1), which indicates complete mixing, and Y_2_ means that the Reynolds number must be maximized, which indicates that the flow characteristics are closely turbulent. The prediction of the optimized vortex tube reactor design parameters is optimized for optimal mixing efficiency.

### 2.6. Fabrication of 3D-Printed Vortex-Tube-Reactor-Based FDM

The fabrication process of the 3D-printed vortex tube reactor was carried out using fused deposition modeling (FDM) technology (UP mini 2, Beijing Tiertime Technology Co., Ltd., Beijing, China) with polypropylene (PP) filament. The design of the vortex tube reactor was created using computer-aided design (CAD) software. The STL file was then converted and sliced into G-code using the slicing software (version no.: 2.6.49.627) of UPstudio (Beijing Tiertime Technology Co., Ltd., Beijing, China), and then loaded into the FDM printer using the printing condition shown in Table 3.

After completing the printing process, the 3D-printed vortex tube reactor was removed from the build platform and cleaned to remove any excess material. In the post-processing stage, dichloromethane was flowed through the reactor for 5 min at a flow rate of 10 mL/min to dissolve any remaining PP and seal any potential leaks. This was followed by a 15 min flow of ethanol at a flow rate of 10 mL/min, which was used to flush out the dichloromethane and ensure that the reactor was free of any remaining residue. The 3D-printed vortex tube reactor was then assembled and tested to ensure its functionality.

### 2.7. Dispersion Experiment

A dispersion experiment was conducted in the optimized reactor to observe the effect of fluid dynamics on the dispersion behavior of the tracer within the reactor. A total of 0.5 mL of 1% (*w*/*v*) erythrosine solution, as a tracer, was injected into the reactor with a flow rate of 10–120 mL/min of DI water. Samples were collected at regular time intervals and the absorbance was measured using a UV-VIS spectrophotometer (2J1-0004, Hitachi, Japan) at a wavelength of 530 nm. The absorbance measurements of tracer concentration (C(t)) at the time (t) were used to evaluate the residence time distribution function $E\left(t\right)$, the mean residence time ($t_{m}$), and the distribution variance ($\sigma^{2}$).

The residence time distribution function (RTD) or $E\left(t\right)$ function can be calculated from the sample data collected at various time intervals. The RTD is the fraction of total fluid in the reactor that leaves at a specific time.

(7) $$E\left(t\right)=\frac{C(t)}{\int_{0}^{\infty }C\left(t\right)dt}$$

The mean residence time ($t_{m}$) can then be calculated from the RTD by finding the first moment of the RTD:

(8) $$t_{m}=\frac{\int_{0}^{\infty }t\cdot C\left(t\right)dt}{\int_{0}^{\infty }C\left(t\right)dt}$$

The distribution variance can also be calculated from the RTD as the second moment of the RTD:

(9) $$\sigma^{2}=\frac{\int_{0}^{\infty }{\left(t-t_{m}\right)}^{2}\cdot C\left(t\right)dt}{\int_{0}^{\infty }C\left(t\right)dt}=\int_{0}^{\infty }t^{2}E\left(t\right)dt-t_{m}^{2}$$

To facilitate a comparison of the results obtained from different flow rates of the vortex tube reactor configurations, it is necessary to use dimensionless units. The dimensionless concentration ($C_{\theta }$) can be calculated by dividing the tracer concentration at time ($C\left(t\right)$) by the initial tracer concentration ($C_{0}$):

(10) $$C_{\theta }=\frac{C\left(t\right)}{C_{0}}$$

Similarly, the dimensionless time (θ) can be calculated by dividing time (*t*) by the mean residence time ($t_{m}$):

(11) $$\theta =\frac{t}{t_{m}}$$

Finally, the dimensionless $E\left(\theta \right)$ can be obtained using $C_{\theta }$ and θ:

(12) $$E\left(\theta \right)=\frac{C(\theta )}{\int_{0}^{\infty }C\left(\theta \right)d\theta }$$

### 2.8. Synthesis and Evaluation of PGA Nanoparticles

**Figure 3:**
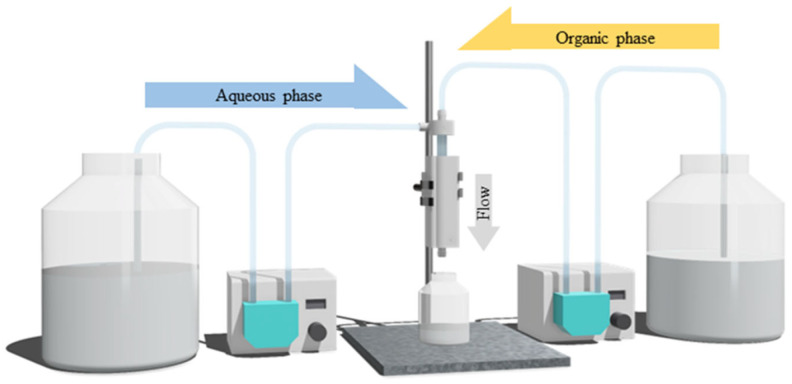
Schematic representation of the experimental set-up of the vortex tube reactor in the synthesis of PGA nanoparticles.

PGA nanoparticles were synthesized using a vortex tube reactor by combining an aqueous solution of 0.5% *w*/*v* of PVA in DI water and an organic phase, which contained 0.5% *w*/*v* PGA in acetone. The impact of different system parameters on the size and PDI of the final formulation was studied by adjusting the total flow rate and aqueous-to-organic volumetric ratio of the incoming streams from 68.92 to 140 mL/min and from a ratio of 3:1 to 9:1 (aqueous-to-organic volumetric ratio), respectively. The total flow rate represents the combined flow rate of the organic and aqueous phases that are pumped through the two inlets, while the aqueous-to-organic volumetric ratio refers to the volume ratio of the aqueous and organic phases, whereby the experimental set-up is shown in Figure 3.

### 2.9. The Physical Characteristics of PGA Nanoparticles

The particles’ size and polydispersity index were evaluated using a Zetasizer (MAL1070387, Malvern, UK). To perform the measurements, the PGA nanoparticles were diluted to a concentration of 0.1% *w*/*v* using deionized water and agitated for 3 min before analysis. The average values and standard error were calculated based on measurements from three batches of samples. Additionally, the surface morphology of the PGA nanoparticles was examined using scanning electron microscopy (SEM) (LEO 1450 VP, Carl Zeiss, Germany). The samples were concentrated via centrifugal ultrafiltration (MWCO 30 kDa, Amicon^®^Ultra-15 filter, Merck, Germany) at 3000 rpm for 20 min. A small amount of the sample was fixed on an SEM stub using double-sided adhesive tape and coated with gold. After thorough drying, the samples were observed using SEM.

### 2.10. Statistical Analysis

The correlations of CFD parameters were determined using Design Expert 11 (Stat-Ease, Inc., Minneapolis, MN, USA) for statistical analysis and graphing of the model response surface. The experimental data were reported as the mean ± standard error of three replicates. To identify statistically significant variations (*p* < 0.05), ANOVA and Levene’s test for homogeneity of variance were conducted using SPSS version 10.0 for Windows (SPSS Inc., New York, NY, USA). For post hoc testing of multiple comparisons (*p* < 0.05), either the Scheffé or Games–Howell test was employed, depending on the significance of Levene’s test.

## 3. Results and Discussion

### 3.1. CFD Mesh Sensitivity

**Figure 4:**
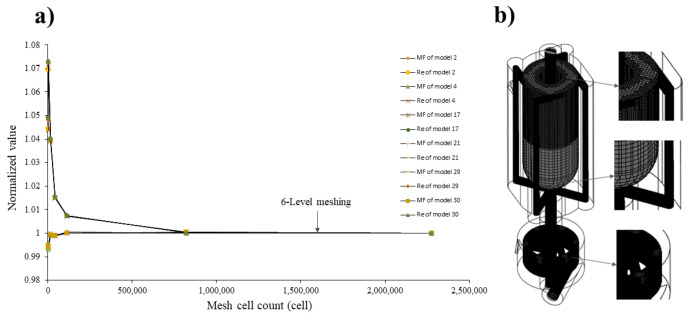
(**a**) Mesh independence test using the vortex tube reactor and (**b**) the automated generated mesh for all vortex tube reactor models studied.

An unstructured and uniform mesh with tetrahedral cells and hexahrdral cells was employed to assess the sensitivity of the outcomes regarding mesh refinement and to determine the optimal cell size in the vortex tube reactor while ensuring accuracy and reliable results in the numerical simulations. The mesh convergence was evaluated by testing seven levels of automated meshing, as shown in Figure 4a, and the normalized value was used to determine the proportion of each level value divided by the 7th level value. The results show that the meshing of 6th level value, as illustrated by the center-point models shown in Figure 4b, initially indicates adequate convergence for obtaining precise outcomes, with a relative error with the 7th level value less than 0.2689% for the mass fraction of water and 1.4667% for the Reynolds number, as shown in Table 4. Other models initially showed convergence that was the same as for the 6th level value, as described in Supplementary Materials Table S3. Therefore, this meshing was chosen as the optimal grid for the study. Based on the findings, the 6th level value offers a balance between accuracy and computational efficiency.

### 3.2. Experimental Design of Vortex Tube Reactor

The results of the central composite design experiments involving four factors, as seen in Table 5, were assessed, and the correlations between independent and dependent variables were analyzed using partial least square regression. The relationship findings between the independent factors (X) and the responses (Y) are presented in Table 6. The PLS model based on the coded factors of MI and Reynolds number was found to be highly significant, with a *p*-value of 0.0003 and the other value being less than 0.0001. This indicates that the model can effectively explain the relationship between the independent variables and the dependent variable.

#### 3.2.1. Mixing Performances of Vortex Tube Reactor (Y_1_)

**Figure 5:**
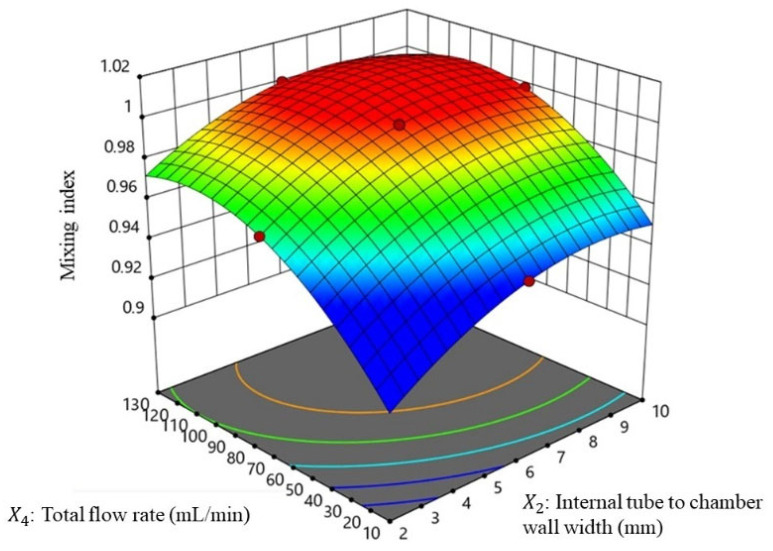
Response surface of the mixing index with the major factor of the total flow rate and the internal tube to chamber wall width.

The mixing index refers to a quantitative measure of how well their substances are mixed homogeneously. It is often used to evaluate the efficiency of a mixing performance in the vortex tube reactor model. The results of the PLS model for predicting the mixing index in this system provide valuable insights into the relative importance of the different factors that affect mixing, while the RS plot of major factors is shown in Figure 5. The coefficients of the PLS equation reveal the relative importance of each variable in predicting the mixing index. The highest positive coefficients were found for $X_{1}^{2}X_{2}^{2}$ (*p* < 0.0001 *), $X_{2}$ (*p* < 0.0001 *), and $X_{4}$ (*p* < 0.0001 *), indicating that these variables have the greatest positive influence on the mixing index. Conversely, the highest negative coefficients were found for $X_{1}^{2}X_{2}$ (*p* < 0.0001 *), $X_{1}^{2}X_{4}$ (*p* < 0.0001 *), $X_{2}^{2}$ (*p* < 0.0001 *), and $X_{4}^{2}$ (*p* < 0.0001 *), indicating that these variables have a negative impact on the mixing index. Other coefficients were found to have smaller effects on the mixing index. These factors may be less critical for predicting the mixing performance, and their influence may be overshadowed by the larger effects of other factors. In the PLS model for $Y_{1}$, the factors of $X_{1}$, $X_{2}$, and $X_{4}$ have positive and negative coefficients in different terms of PLS model, which suggests that increasing these factors can lead to better mixing performance, but extreme values of these factors can reduce the mixing index. This finding is supported by previous studies on the design of the passive mixer of threaded inserts for enhancing the mixing performance via flow chemistry. The results showed that the design of the flow direction via threaded insert for vortex streamlines and increasing the Reynolds number by increasing the flow rate had the greatest impact on mixing performance [11].

The findings of this study are consistent with the Reynolds transport theorem, which suggests that increasing the diameter of the reactor results in a more turbulent flow of the fluid [20,21]. This increased turbulence can lead to better mixing efficiency, as the fluid experiences greater mixing and agitation. The study also found that increasing the flow rate resulted in the formation of vortex streamlines in the reactor, which further enhanced the mixing efficiency. Overall, these findings can be useful for the design and optimization of similar systems, as they provide insights into the factors that can have the greatest impact on mixing performance. The high significance and accuracy of the PLS model also suggest that it can be a valuable tool for predicting mixing performance in this system.

#### 3.2.2. Flow Structures of Vortex Tube Reactor (Y_2_)

**Figure 6:**
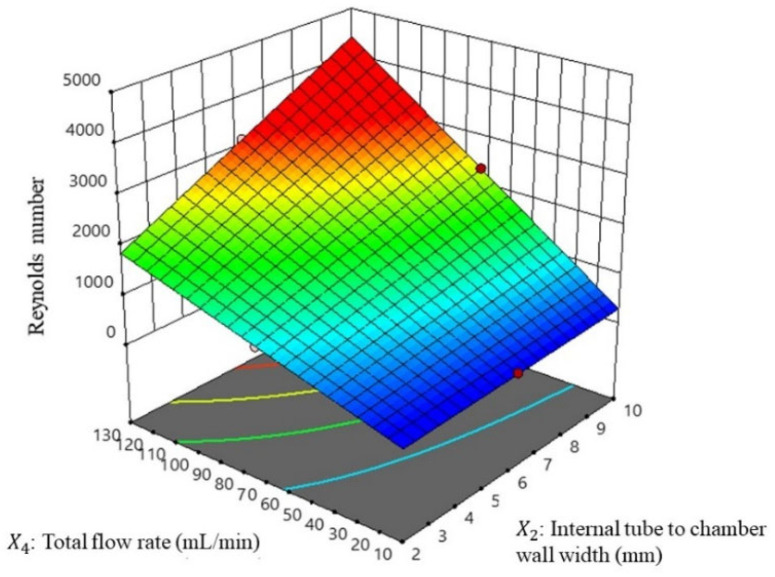
Response surface of the Reynolds number with the major factor of the total flow rate and the internal tube to chamber wall width.

Reynolds number analysis is used to determine the flow regime of a fluid and predict the flow behavior within the vortex tube reactor model based on its velocity, density, viscosity, and characteristic length. Reynolds number (Re) is a dimensionless quantity that helps to classify the type of flow, with regard to whether it is laminar, transitional, or turbulent. In this study, the coefficients of the PLS equation reveal the relative importance of each variable in predicting the Reynolds number, while the RS plot of major factors is shown in Figure 6. The highest positive coefficient was found for $X_{2}X_{4}$ (*p* < 0.0001 *), $X_{2}$ (*p* < 0.0001 *), and $X_{4}$ (*p* < 0.0001 *), indicating the greatest positive influence on the Reynolds number. On the other hand, the coefficient for $X_{1}$ (*p* = 0.0005 *) was found to have a negative impact on the Reynolds number. This may suggest that increasing the reactor diameter, increasing the flow rate, and decreasing the reactor length could result in higher Reynolds numbers, which relates to the Reynolds transport theorem.

This study found a positive correlation between the increase in the Reynolds number and improved mixing performance in the vortex tube reactor. However, increasing the reactor length can reduce the Reynolds number. This is because a longer reactor allows for more time for the fluid to develop a more steady and consistent flow, which can reduce turbulence and fluctuations in the fluid flow [22,23].

Overall, these findings can be useful for the design and optimization of similar systems, as they provide insights into the factors that can have the greatest impact on the Reynolds number. The high significance and accuracy of the PLS model also suggest that it can be a valuable tool for predicting Reynolds numbers in this system.

### 3.3. OptiMized Vortex Tube Reactor

Once the experimental design was completed, partial least squares regression models and response surfaces were generated, as well as the constraints for all factors, and the desirability of all responses was determined, as presented in Table 2. The optimization of the vortex tube reactor was performed and the prediction values of the factors $X_{1}$, $X_{2}$, $X_{3}$, and $X_{4}$ were obtained as 79.81 mm, 8.95 mm, 86.74 mm, and 104.46 mL/min, respectively. The vortex tube reactor was prepared and CFD-analyzed according to the optimized conditions. Table 7 shows the prediction ability percentages obtained by comparing the predicted responses from the partial least squares regression models with the actual test values. The actual values were very close to the predicted values. For the response variables, the prediction abilities of $Y_{1}$ and $Y_{2}$ were found to be 99.95% and 100.15%, respectively, which provided a desirability value of 1. The high percentage of prediction ability for both of the factors and the response variables suggests that the optimization process was successful in predicting the optimal values for the factors and achieving the desired responses. These results indicate that the optimized conditions of the vortex tube reactor can lead to improved mixing performance and Reynolds number.

#### 3.3.1. Mixing Performance Evaluation

**Figure 7:**
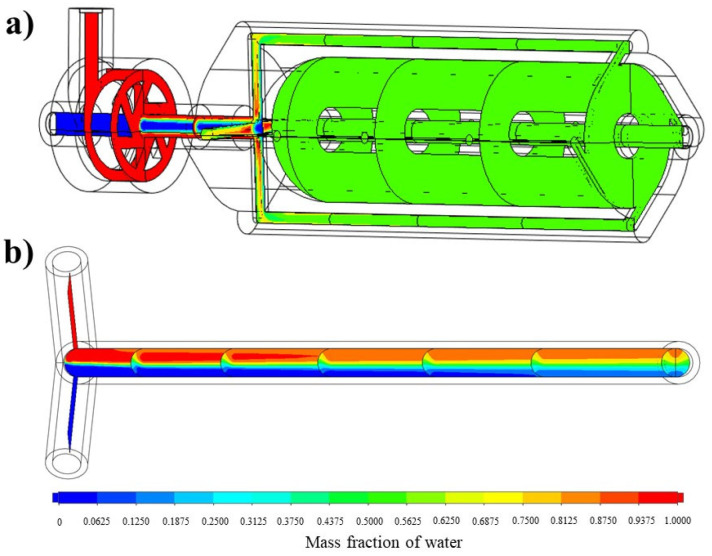
The mass fraction cut plot of (**a**) the vortex tube reactor and (**b**) the tubular reactor with 4 mm diameter.

**Figure 8:**
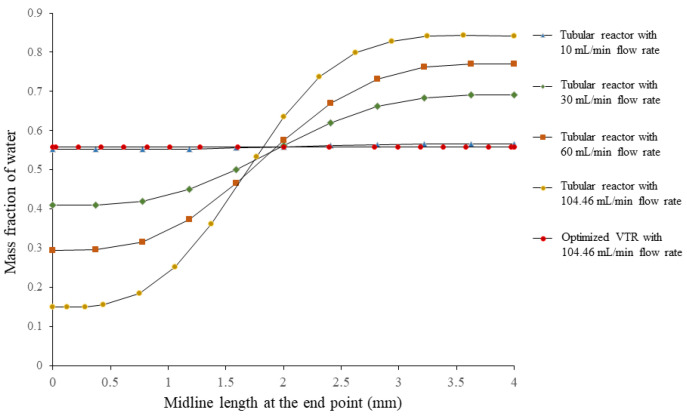
Mass fraction evolutions along the midline at the exit of the optimized vortex tube reactor model and the tubular reactor with different flow rates.

The analysis of the mixing performance of the optimized vortex tube reactor was performed via CFD. The result showed that the mixing index was 0.99948, indicating a homogeneous concentration and great mixing performance (MI approached 1 and σ approached 0, indicating perfect mixing) [12]. The mass fraction cut plot of the vortex tube reactor (Figure 7a) showed that the fins on the mixer helped to accelerate the mixing process, and when the fluid flowed into the by-pass channels, mixing occurred almost completely before returning to the vortex chamber and exiting through the outlet. Figure 7a shows a color change in the mass fraction of water in the optimized vortex tube reactor from red (high mass fraction of water) and blue (low mass fraction of water) to green (steady mass fraction of water). The measurement of mass fraction evolutions along the midline at the exit of the vortex tube reactor (Figure 8) revealed that these profiles were almost similar along the Y-direction of the graph, with a standard deviation value of only 0.00026, confirming the homogeneity of the mixing.

#### 3.3.2. Reynolds Number Evaluation

**Figure 9:**
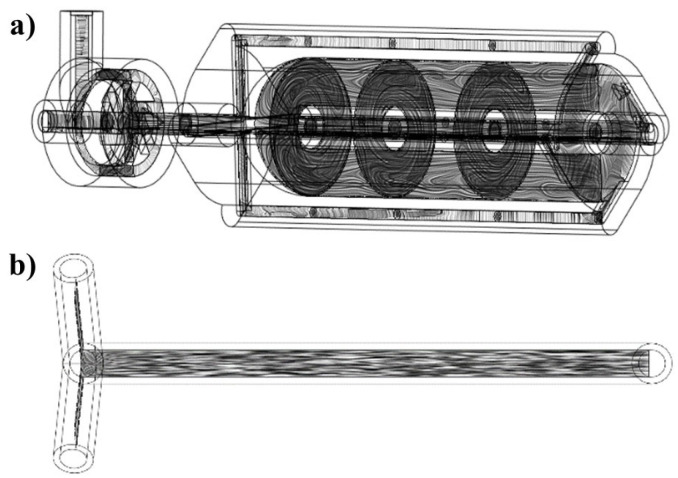
The vorticity streamline cut plot of (**a**) the vortex tube reactor and (**b**) the tubular reactor with 4 mm diameter.

The purpose of conducting a Reynolds number analysis is to identify the flow regime of a fluid and make predictions about the flow behavior within the optimized vortex tube reactor. The analysis of the Reynolds number revealed that the value was 3240.55, indicating that the flow characteristics of the optimized vortex tube reactor were in a transitional state (2500 < *Re* < 4000) [14]. This result is consistent with the vorticity streamline cut plot shown in Figure 9a, where the streamlines inside the vortex tube reactor have a swirling and eddying pattern, as well as some laminar patterns. This observation further confirms the transitional flow regime, which is characterized by the coexistence of both laminar and turbulent flow patterns. This finding is supported by previous studies on the transitional flow regime in various types of reactors. The transitional flow regime is known to be associated with enhanced mixing and improved mass transfer due to the coexistence of both laminar and turbulent flow patterns. Therefore, the transitional flow regime in the optimized vortex tube reactor is expected to contribute to the improved mixing performance observed in this study [13].

#### 3.3.3. Relationship of Mixing Index with Different Flow Rates

**Figure 10:**
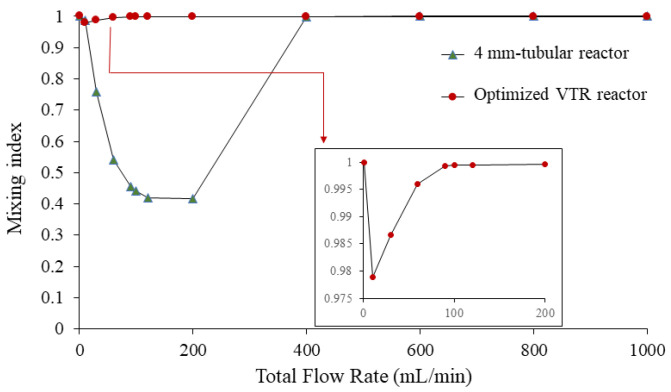
The mixing index at different flow rates of the vortex tube reactor and the tubular reactor with 4 mm diameter.

The mixing index (MI) at different flow rates is presented in Figure 10. The increase in flow rate leads to an increase in Reynolds number, indicating a transition from laminar to turbulent flow. However, the mixing index initially remains high, indicating good mixing at low flow rates, dominated by the diffusion term. In addition, the observation that the mixing index initially remains high at low flow rates is consistent with the theoretical expectation that diffusion dominates at low Reynolds numbers [24].

At a flow rate of 10 mL/min, corresponding to a Reynolds number of 310.21, the mixing index started to slightly decrease. This observation suggests that the onset of turbulence occurs and the increase in the convection term is not strong enough to fully homogenize the fluid. Therefore, slightly increased turbulence may not be able to achieve complete mixing, leading to a reduction in the mixing index. This phenomenon has been previously reported in the literature, where it has been shown that the transition from laminar to turbulent flow can result in incomplete mixing due to the presence of stagnant regions and the difficulty of stirring the fluid in these regions [25].

For flow rates higher than 10 mL/min, the mixing index tends to increase and remains relatively stable at flow rates higher than 90 mL/min. This observation indicates that the convection term plays a role in increasing the mixing performance of the vortex tube reactor at Reynolds numbers higher than 310.21. Hence, the vortex tube reactor is very effective at mixing with a wide range of flow rates.

We performed a comparison of a vortex tube reactor and tubular reactor with a diameter of 4 mm connected to a Y-mixer. The simulation of the tubular reactor was performed under the same volume and boundary conditions as the optimized vortex tube reactor; the mixing index of the tubular reactor was found to be 0.392088, seen in the mass fraction cut plot shown in Figure 7b. The mass fraction evolutions along the midline at the exit of the typical tubular reactor showed an S-curve along the Y-direction of the graph (Figure 8), with a larger standard deviation value of 0.303952, indicating an unmixed state at the exit. The Reynolds number was measured to be 499.07, indicating laminar flow in the tubular reactor (Figure 9b) [14].

When the flow rate in a tubular reactor drops below 10 mL/min or exceeds 400 mL/min (Figure 10), the mixing index tends to increase. However, the variance in mass fraction along the midline at the exit of the reactor decreases (Figure 8). This trend continues until the flow rate drops below 10 mL/min or exceeds 400 mL/min, and at which point the mixing index is no longer significantly different (*p*-value = 0.1733) from that of the vortex tube reactor at any flow rate. However, increasing the diameter or flow rate of the tubular reactor is not recommended as this would reduce the mixing performance and productivity. The reactor length should be sufficient to achieve complete mixing, or the flow rate should be slow enough to ensure good mixing without sacrificing productivity. While high flow rates may increase the mixing index, a high pressure drop can negatively affect the system’s efficiency by increasing energy consumption and reducing flow rates. A high pressure drop requires more energy to maintain the desired flow rate, limiting the overall efficiency of the system. Thus, the vortex tube reactor is a promising solution as it offers better mixing performance with a smaller reactor size and increased flow rate, which can enhance productivity. It offers a wide range of flow rate adjustments while maintaining homogeneous mixing. This is consistent with previous studies that have shown the effectiveness of vortex tube reactors in achieving higher mixing performance and productivity compared to traditional tubular reactors.

### 3.4. Fabrication of Vortex-Tube-Reactor-Based FDM

In this study, we successfully fabricated a vortex tube reactor using the fused deposition modeling (FDM) 3D printing technique. The reactor was designed with optimized parameters. The material used for printing was PP, which has good thermal and mechanical properties suitable for this application.

**Figure 11:**
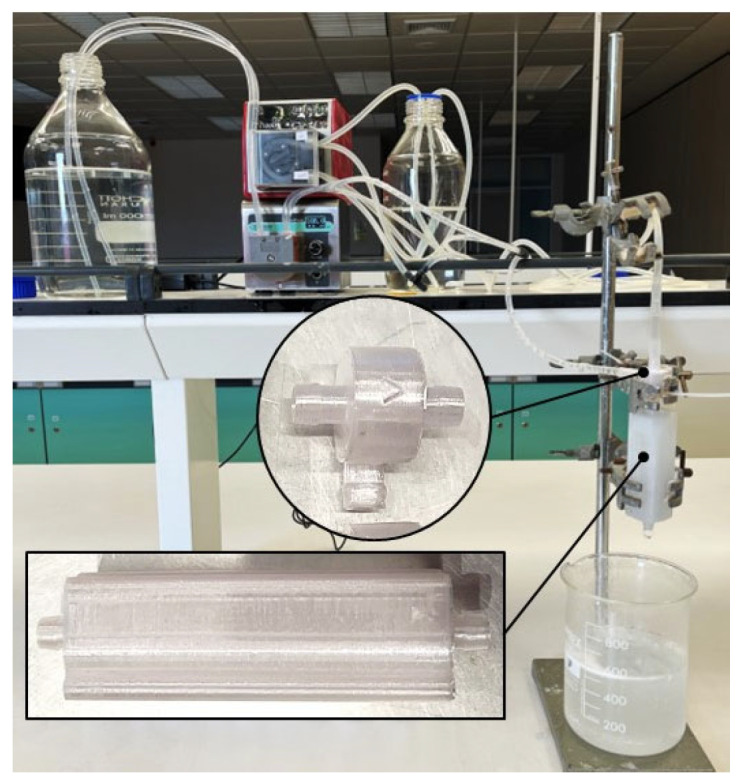
Schematic representation of the 3D-printed vortex tube reactor and assembly.

The fabrication process involved slicing the 3D model into layers and printing them using a desktop 3D printer. The printing parameters were optimized to obtain a smooth and defect-free surface. The 3D-printed vortex tube reactor and assembly are shown in Figure 11.

### 3.5. Dispersion Experiment

**Figure 12:**
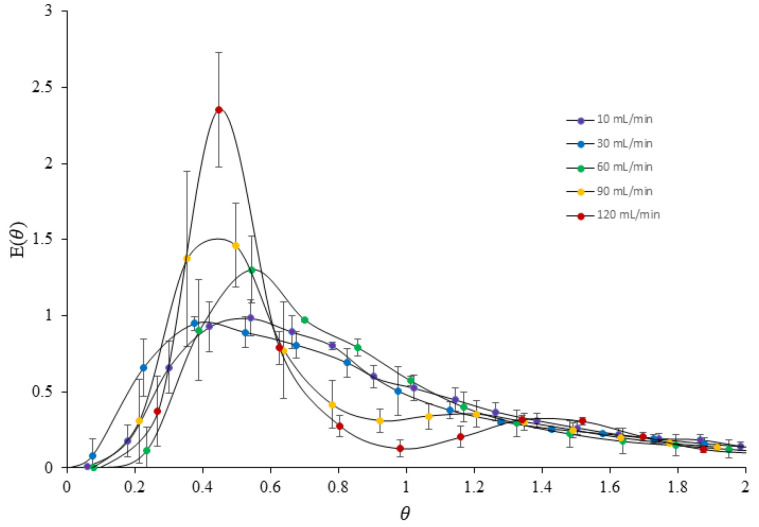
Comparison of E(*θ*) curves in different flow rates of the printed vortex tube reactor.

The residence time distribution (RTD) is an important characteristic of chemical reactors, providing a quantitative measure of the degree of back mixing within the system. The RTD of a vortex tube reactor was determined using erythrosine as a tracer in a series of experiments conducted at different flow rates. The E(*θ*) curves obtained from the experiments revealed significant differences in flow patterns with changing flow rates, as shown in Figure 12. At low flow rates of 10–60 mL/min, a broad E(*θ*) curve was observed, indicating a skewed distribution with a shorter mean residence time and some degree of back mixing inside the reactor. The E(*θ*) curves also showed a long tail of tracer, suggesting a wide range of residence times in the system. In contrast, at higher flow rates of 90–120 mL/min, the flow pattern was significantly narrower with a bimodal distribution, indicating a more uniform and less dispersed flow through the reactor vessel. An increase in flow rate led to an increase in the symmetry of the RTD curves, indicating the minimization of disturbances. However, all of the peaks of the E(*θ*) curves for different flow rates showed a value of *θ* lower than 1, indicating that most of the tracers spent less time in the reactor than the mean residence time. These findings suggest that the vortex tube reactor exhibits passive mixing and enhanced mixing efficiency with an increased flow rate [26].

The results of the experiments also indicated that the degree of back mixing decreased with an increased flow rate due to increasing turbulence inside the reactor, which helped to enhance mass transfer and improve mixing efficiency. Table 8 shows that the decreasing value of the distribution variance (σ^2^) at higher flow rates indicated that the tracer flow through the vortex tube reactor was more homogeneous, making it closer to an ideal plug flow reactor. This result is consistent with computational fluid dynamics (CFD) simulations of the reactor, which have shown that an increased flow rate can enhance mixing efficiency.

However, the E(*θ*) curve was unable to fit the axial dispersion model (ADM) and the tank in the series model (TIS). This model assumes a uniform flow and concentration profile, which may not be the case in a vortex tube reactor. As a result, the model may not be appropriate for our specific experimental set-up, and it may not be possible to obtain a significant fit to our data using standard fitting techniques. This suggests that the flow and mixing patterns in our system may be more complex than can be captured by the axial dispersion model and the tank in the series model. While we explored alternative models and fitting techniques, we were unable to find a more appropriate model for our system.

### 3.6. Formulation of PGA Nanoparticles

In this section, the formulation of PGA nanoparticles is examined, focusing on the influence factor of input flow parameters, which consist of the total flow rate and the aqueous-to-organic volumetric ratio, on characteristics and performance. Additionally, a comprehensive comparison is conducted among PGA nanoparticles synthesized using three different methods, evaluating their respective advantages, limitations, and overall effectiveness.

#### 3.6.1. Effect of Input Flow Rate on Formulated PGA Nanoparticles

**Figure 13:**
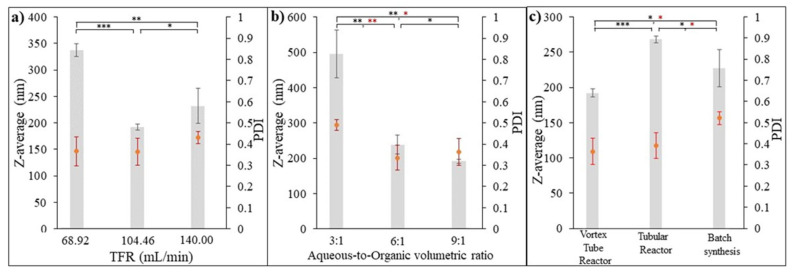
Z-average and PDI analysis of formulated PGA nanoparticles. (**a**) Effect of input flow parameters, (**b**) effect of aqueous-to-organic volumetric ratio, and (**c**) comparison of formulated PGA nanoparticles using three different synthesis methods. Error bar represents standard error of mean. (n = 3, * *p*-value < 0.05, ** *p*-value < 0.01, and *** *p*-value < 0.001.)

The aim of this test was to investigate the effect of input flow rate on the synthesis of PGA nanoparticles using a 3D-printed vortex tube reactor approach. The organic phase, consisting of 0.5% *w*/*v* PGA in acetone, was combined with an aqueous phase containing 0.5% *w*/*v* PVA in DI water, with a fixed ratio of 9:1 (aqueous phase/organic phase). The total flow rate was varied to assess its impact on the nanoparticle characteristics. Figure 13a illustrates the impact of varying the total flow rate on the characteristics of the formulated PGA nanoparticles. At a total flow rate of 68.92 mL/min, the highest z-average particle size was measured as being 337.03 ± 6.3983 nm with a PDI value of 0.366 ± 0.035. Conversely, the lowest z-average particle size of 191.83 ± 2.95 nm and a PDI value of 0.365 ± 0.321 were obtained at a flow rate of 104.46 mL/min. Intriguingly, at a higher flow rate of 140.00 mL/min, the z-average particle size increased once again to 232.00 ± 17.05 nm, while the PDI value reached 0.431 ± 0.014.

This observation suggests that there is an optimal flow rate of 104.46 mL/min for obtaining PGA nanoparticles with a desired particle size with a more uniform size distribution. This flow rate allows for optimal mixing and reaction conditions, leading to better control over particle formation and the ability to minimize size variations. Higher flow rates result in shorter residence time, reducing the time available for nucleation and the growth of nanoparticles. Lower flow rates, on the other hand, provide longer residence time, allowing for more significant particle growth and larger sizes. Flow rates higher than the optimal one can lead to incomplete reactions and particle agglomeration, preventing particles from reaching their desired size and causing the formation of larger aggregates. This agglomeration is more likely at higher flow rates due to increased collision frequency between particles, favoring agglomeration over individual growth [27].

Overall, these results demonstrate the significant influence of input flow parameters, specifically the total flow rate, on the characteristics of the formulated PGA nanoparticles. It is important to carefully optimize the flow rate to achieve the desired particle size and size distribution.

#### 3.6.2. Effect of Aqueous-to-Organic Volumetric Ratio on Formulated PGA Nanoparticles

To further investigate the influence of process parameters on the synthesis of PGA nanoparticles using flow chemistry, the volumetric ratio of the aqueous phase to the organic phase was varied while keeping the total flow rate constant at 104.46 mL/min. The organic phase consisted of 0.5% *w*/*v* PGA in acetone, while the aqueous phase contained 0.5% *w*/*v* PVA in DI water. The z-average particle size (nm) and polydispersity index (PDI) were measured to assess the impact of the ratio on particle characteristics. Figure 13b illustrates the impact of the aqueous-to-organic volumetric ratio on the characteristics of the PGA nanoparticles that were formulated. By manipulating this ratio, variations in particle sizes were observed. When the ratio was set at 3:1 (aqueous-to-organic volumetric ratio), the z-average particle size was measured as being 496.20 ± 34.13 nm, and the PDI value was 0.491 ± 0.013. These results indicate a larger particle size and wider size distribution compared to the other ratios tested. However, as the ratio increased to 6:1 and 9:1, the z-average particle size decreased to 239.17 ± 13.45 nm and 191.83 ± 2.95 nm, respectively. Furthermore, the corresponding PDI values decreased to 0.337 ± 0.030 and 0.364 ± 0.032, respectively. These findings suggest that increasing the proportion of the aqueous phase relative to the organic phase leads to smaller PGA nanoparticles and promotes a more uniform distribution of particle sizes. Increasing the aqueous phase ratio (i.e., increasing the proportion of the aqueous phase relative to the organic phase) generally results in improved particle size and size distribution uniformity for formulated PGA nanoparticles. This behavior can be attributed to the role of the aqueous phase, specifically the presence of polyvinyl alcohol (PVA), in the synthesis process. This might be because PVA acts as a stabilizer or surfactant in nanoparticle synthesis. It aids in controlling the growth and agglomeration of particles, leading to smaller and more uniformly dispersed nanoparticles. As the aqueous phase ratio increases, there is a higher concentration of PVA in the reaction mixture. This increased concentration of PVA helps to stabilize the PGA nanoparticles and inhibit particle growth or aggregation, resulting in smaller particle sizes. Conversely, a lower aqueous phase ratio (higher organic phase ratio) may limit the availability of PVA in the reaction mixture, leading to less effective stabilization and allowing for increased particle growth or aggregation. This can result in larger particle sizes [28,29]. The results highlight the significance of the aqueous phase to organic phase ratio in controlling the particle size and size distribution of formulated PGA nanoparticles. Adjusting this ratio provides a means to tailor the particle characteristics according to the desired application requirements. Further optimization of the ratio, as well as investigating a wider range of ratios, could be explored to achieve precise control over particle size and size distribution.

#### 3.6.3. Comparison of the Formulated PGA Nanoparticles Using Three Different Synthesis Methods

The PGA nanoparticles were synthesized using three different synthesis methods which were as follows: (i) the optimized design 3D-printed vortex tube reactor, (ii) the tubular reactor with 4 mm diameter and 80 cm length, operating under the same flow system condition as (i) with a total flow rate of 104.46 mL/min and an optimal aqueous-to-organic volumetric ratio, and (iii) batch synthesis using the emulsification solvent evaporation method for 12 h with the same aqueous phase to organic phase ratio. The size and size distribution comparison depicted in Figure 13c demonstrate that the 3D-printed vortex tube reactor yields the smallest z-average values when compared to the tubular reactor (*p*-value < 0.001) and batch synthesis methods (*p*-value < 0.01). Specifically, the 3D-printed vortex tube reactor exhibits z-average particle sizes of 191.83 ± 2.95 nm. In contrast, the tubular reactor and batch synthesis methods result in larger particle sizes of 267.33 ± 2.50 nm and 226.7 ± 13.20 nm, respectively. For PDI values, the 3D-printed vortex tube reactor and tubular reactor exhibit lower PDI values compared to the batch synthesis approach. The 3D-printed vortex tube reactor demonstrates a PDI value of 0.3643 ± 0.0321, while the tubular reactor shows a PDI value of 0.3913 ± 0.0531. On the other hand, the batch synthesis method has a higher PDI value of 0.521 ± 0.016. These results suggest that the utilization of the 3D-printed vortex tube reactor and tubular reactor demonstrated a significant advantage over the batch synthesis method, as they yielded a notably more uniform particle size distribution. Moreover, both flow chemistry systems exhibited a remarkable ability to achieve fast and homogeneous mixing of reactants, which is particularly advantageous for sensitive and rapid nano-precipitation reactions [30].

**Figure 14:**
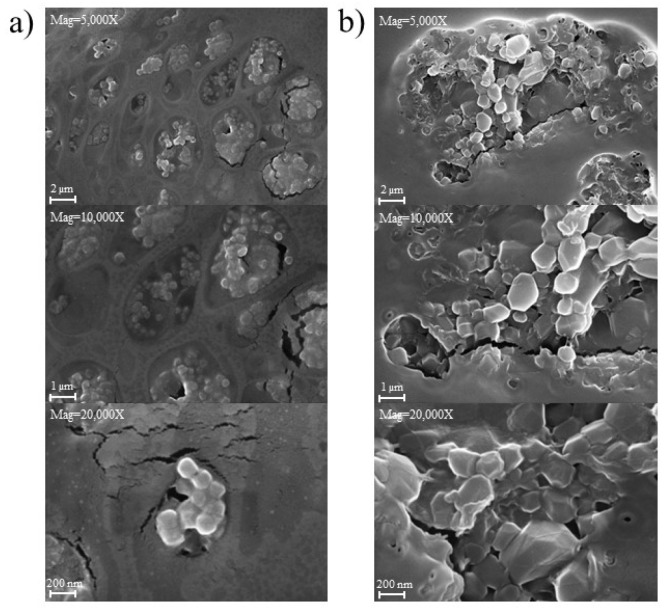
SEM images of PGA nanoparticles prepared using (**a**) 3D-printed vortex tube reactor and (**b**) tubular reactor.

Figure 14 displays the morphology of PGA nanoparticles prepared using both the 3D-printed vortex tube reactor (Figure 14a) and the tubular reactor (Figure 14b). The SEM images revealed that the nanoparticles prepared from both methods exhibited a spherical shape, smooth surface, and agglomeration, forming clusters with nanoparticles sticking together. Interestingly, the nanoparticles produced by the 3D-printed vortex tube reactor exhibited a smaller and more uniform size compared to those prepared by the tubular reactor. The PGA particle size from the 3D-printed vortex tube reactor measured from SEM images were from 292.2 to 324.0 nm, while some particles in the samples from the tubular reactor reached dimensions of up to 725.8 nm. This observation is consistent with the dynamic light scattering data (Figure 13c), despite the fact that the SEM particle size measurements indicated a larger particle size than the dynamic light scattering method, possibly as a result of sample preparation. This might be due to the drying and fixation processes on the solid substrate during the SEM sample preparation. Some dispersed PVA might coat and aggregate the PGA nanoparticles, leading to particle aggregation or changes in particle size and shape [31].

The 3D-printed vortex tube reactor demonstrates clear advantages over both the 4 mm diameter tubular reactor and the batch synthesis method in terms of particle size and size distribution. The 3D-printed vortex tube reactor’s ability to consistently produce smaller and more uniform nanoparticles suggests higher productivity compared to the other methods. By achieving a narrower size distribution and smaller particle sizes, the 3D-printed vortex tube reactor enables the synthesis of nanoparticles with desired characteristics, making them suitable for a wide range of applications. Consequently, the enhanced productivity of the 3D-printed vortex tube reactor in generating nanoparticles with favorable properties reinforces its potential as an efficient and reliable method for nanoparticle synthesis.

## 4. Conclusions

The study successfully investigated the mixing performance and flow characteristics of the vortex tube reactor through CFD simulations, experimental design, and optimization. The findings provide valuable insights into the factors affecting mixing index and Reynolds number in the vortex tube reactor system. The results can serve as a foundation for designing and optimizing similar systems, while the PLS models offer a reliable tool for predicting mixing performance and Reynolds numbers. The study also revealed that the optimized vortex tube reactor exhibits excellent mixing performance, characterized by a high mixing index and a transitional flow regime. This reactor allows for a wide range of flow rate adjustments, and the passive mixing from the RTD function of the 3D-printed vortex tube reactor further enhances the mixing performance. These findings demonstrate the significant potential of the new design of the 3D-printed vortex tube reactor to improve particle size, PDI, and productivity in PGA nanoparticle synthesis, surpassing traditional methods. Future studies can build upon these findings for other nanoparticle drug delivery systems such as inorganic nanoparticles, liposomes, or solid–lipid nanoparticles.

## Figures and Tables

**Table 1:** Factor levels and response based on central composite design.

Factor (X)	Unit	DoE Code				
		−α	−1	0	1	+α
X_1_: Reactor length	mm	10	30	45	60	90
X_2_: Internal tube to chamber wall width	mm	2	4	6	8	10
X_3_: Internal tube length per reactor length (%)	%	10	30	45	60	90
X_4_: Total flow rate	mL/min	10	40	70	100	130

**Table 2:** Constraints and criteria of the optimized vortex tube reactor model.

	Factor (X) and Response (Y)	Constraint	Criteria
X_1_	Reactor length	10 < X_1_ < 90	in range
X_2_	Internal tube to chamber wall width	2 < X_2_ < 10	in range
X_3_	Percentage of internal tube length/reactor length	10 < X_3_ < 90	in range
X_4_	Total flow rate	10 < X_4_ < 130	in range
Y_1_	Mixing index	0.947361 < Y_1_ < 1	target = 1
Y_2_	Reynolds number	239.45 < Y_2_ < 3132.25	maximize

**Table 3:** The FDM 3D printing condition.

Printing Condition	Parameter Setting
Polymer	PP Filament
Nozzle Diameter	0.4 mm
Layer Height	0.2 mm
Print Speed	50 mm/s
Infill	20%
Print Temperature	210 °C
Bed Temperature	50 °C

**Table 4:** Relative error with the highest-level automated generated grids (the 7th level).

Run	Mesh No. of 6th Level Meshing (Cells)	Iteration	%Relative Error with 7th Level	
			Mass Fraction of Water	Reynolds Number
1	817,569	521	0.0537	0.0785
2	821,352	508	0.0000	0.0417
3	658,343	447	0.0179	0.0749
4	821,325	508	0.0000	0.0417
5	133,316	437	0.0538	0.0732
6	849,119	383	0.1439	0.0677
7	1,031,558	488	0.1436	0.0333
8	1,428,243	526	0.0717	0.0680
9	1,010,688	419	0.1799	0.0942
10	821,352	534	0.0896	0.2214
11	639,576	461	0.2689	0.4146
12	658,350	531	0.0358	0.0438
13	849,077	434	0.0716	0.5331
14	1,032,001	459	0.1076	0.0679
15	821,352	401	0.0000	0.0627
16	1,010,660	449	0.0539	0.3500
17	821,352	506	0.0179	0.0298
18	195,603	275	0.1073	1.4667
19	941,032	584	0.0000	0.0627
20	673,532	437	0.0179	0.0356
21	821,366	506	0.0179	0.0268
22	817,667	636	0.0000	0.0584
23	733,085	518	0.0358	0.3908
24	276,519	307	0.0896	0.1132
25	639,597	381	0.0179	0.0313
26	1,427,656	580	0.0538	0.0313
27	1,013,285	557	0.0179	0.0657
28	630,716	624	0.0179	0.0358
29	821,352	506	0.0358	0.0298
30	821,086	506	0.0179	0.0268

**Table 5:** Experimental runs and response results in the central composite design.

Model	X_1_	X_2_	X_3_	X_4_	Y_1_	Y_2_
1	70	8	70	40	0.9956	1070.28
2	50	6	50	70	0.9978	1677.65
3	70	8	30	40	0.9958	1148.58
4	50	6	50	70	0.9979	1677.65
5	30	8	70	40	0.9928	1148.88
6	30	4	30	40	0.9886	767.20
7	70	4	30	100	0.9989	1924.36
8	70	4	70	40	0.9863	765.72
9	30	4	70	40	0.9899	765.36
10	50	6	50	130	0.9997	3132.25
11	30	8	30	100	0.9995	2911.32
12	70	8	30	100	0.9997	2879.04
13	30	4	30	100	0.9998	1940.40
14	70	4	30	40	0.9943	766.20
15	50	6	50	10	0.9474	239.45
16	30	4	70	100	0.9973	1936.08
17	50	6	50	70	0.9988	1679.05
18	10	6	50	70	0.9974	1753.35
19	90	6	50	70	0.9963	1676.70
20	50	6	10	70	0.9989	1685.70
21	50	6	50	70	0.9983	1679.00
22	70	8	70	100	0.9997	2876.46
23	30	8	70	100	0.9998	2905.50
24	50	2	50	70	0.9668	1007.88
25	30	8	30	40	0.9952	1150.86
26	70	4	70	100	0.9982	1916.36
27	50	6	90	70	0.9957	1676.45
28	50	10	50	70	0.9981	2349.69
29	50	6	50	70	0.9983	1679.05
30	50	6	50	70	0.9983	1679.00

**Table 6:** Partial least square regression model (PLS model) in terms of coded factors.

PLS Models	*p*-Value	R^2^	Adjusted R^2^
$Y_{1}={0.0082X}_{1}^{2}X_{2}^{2}-0.0008X_{1}X_{2}X_{3}X_{4}-{0.0063X}_{1}^{2}X_{2}-\;{0.0097X}_{1}^{2}X_{4}+0.0006X_{1}X_{2}^{2}+{0.0006X}_{1}X_{2}X_{3}-\;0.0001X_{1}X_{2}X_{4}+0.0005X_{1}X_{3}X_{4}-0.0003X_{1}^{2}-0.004X_{2}^{2}-\;0.0002X_{3}^{2}-0.0062X_{4}^{2}+0.0001X_{1}X_{2}-0.0003X_{1}X_{3}-\;0.0003X_{1}X_{4}+0.0005X_{2}X_{3}-0.001X_{2}X_{4}+0.0004X_{3}X_{4}-\;0.0003X_{1}+0.0078X_{2}{-0.0008X}_{3}+{0.0131X}_{4}+0.998$	0.0003 *	0.9998	0.9990
$Y_{2}=150.063X_{2}X_{4}-13.8292X_{1}+333.036X_{2}+728.835X_{4}+1682.18$	<0.0001 *	0.9995	0.9995

* statistical significance at a *p*-value of 0.05.

**Table 7:** Comparison between the predicted values from PLS models and the actual values of the optimized vortex tube reactor model.

Mesh No. (Cells)	Iteration	Factor (X) and Response (Y)	Prediction Values	Actual Values	Percentage of Prediction Ability (%)
880,082	743	X_1_	79.81	-	-
		X_2_	8.95	-	-
		X_3_	86.74	-	-
		X_4_	104.46	-	-
		Y_1_	1.0000	0.9995	99.95
		Y_2_	3244.46	3240.55	99.88

**Table 8:** Mean residence times and distribution variance in the 3D-printed optimized vortex tube reactor for different flow rates.

Flow Rate (mL/Min)	$t_{m}$(s)	$\sigma^{2}$
10	241.27	15,601.41
30	66.48	1787.27
60	26.95	244.34
90	21.12	218.23
120	16.78	146.42

## Data Availability

Not applicable.

## Supplementary Materials

The following supporting information can be downloaded at https://www.mdpi.com/article/10.3390/nano13192679/s1. Table S1: Properties of water and ethanol for CFD calculation from an engineering database (Solidworks); Table S2: Difference in mesh refinement of the automated generated mesh with seven levels; Table S3: Mesh independency test using the vortex tube reactor model.
