# Supplementary material for: Three-Dimensional-Printed Vortex Tube Reactor for Continuous Flow Synthesis of Polyglycolic Acid Nanoparticles with High Productivity
Source: Nanomaterials (Basel). 2023 Sep 29;13(19):2679. doi: 10.3390/nano13192679 (PMC10574274; doi:10.3390/nano13192679)
Supplement: Supplementary file 1 [file nanomaterials-13-02679-s001.zip › nanomaterials-2614080-supplementary.pdf]

*Supplementary Materials*

# Three-Dimensional-Printed Vortex Tube Reactor for Continuous Flow Synthesis of Polyglycolic Acid Nanoparticles with High Productivity

**Kittipat Suwanpitak [1], Pornsak Sriamornsak [2,3,4], Inderbir Singh [5], Tanikan Sangnim [1,*] and Kampanart Huanbutta [6,*]**

1 Faculty of Pharmaceutical Sciences, Burapha University, Chonburi 20131, Thailand; kittipatsuwanpitak@gmail.com
2 Department of Industrial Pharmacy, Faculty of Pharmacy, Silpakorn University, Nakhon Pathom 73000, Thailand; sriamornsak_p@su.ac.th
3 Academy of Science, The Royal Society of Thailand, Bangkok 10300, Thailand
4 Faculty of Pharmaceutical Sciences, Chulalongkorn University, Bangkok 10330, Thailand
5 Chitkara College of Pharmacy, Chitkara University, Patiala 140401, Punjab, India; inderbir.singh@chitkara.edu.in
6 Department of Manufacturing Pharmacy, College of Pharmacy, Rangsit University, Pathum Thani 12000, Thailand
* Correspondence: tanikan@go.buu.ac.th (T.S.); kampanart.h@rsu.ac.th (K.H.)

This file supplementary Table S1-3.

**Table S1.** Properties of water and ethanol for CFD calculation from the engineering database (Solidworks).

| Properties of water | | | |
|---|---|---|---|
| Density | Dynamic viscosity | Specific heat (Cp) | Thermal conductivity |
| 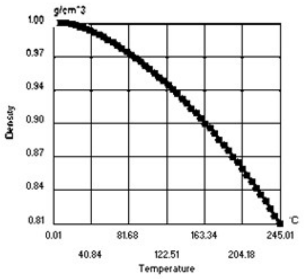  | 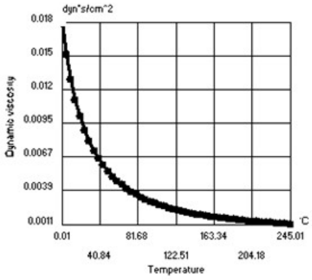  | 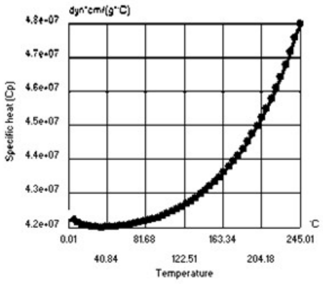  | 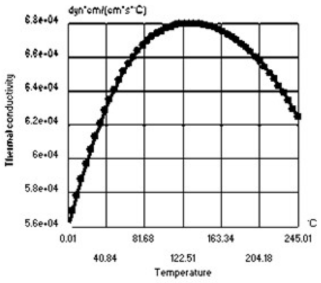  |
| **Properties of ethanol** | | | |
| Density | Dynamic viscosity | Specific heat (Cp) | Thermal conductivity |
| 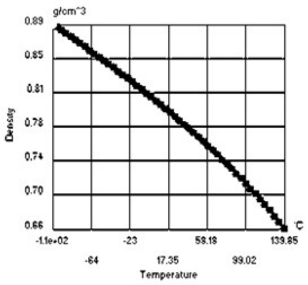  | 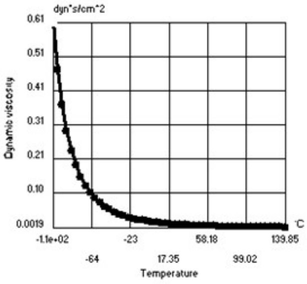  | 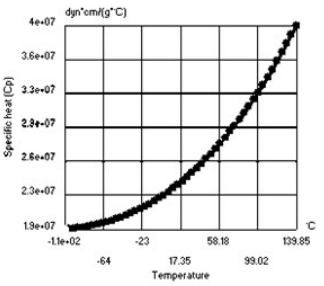  | 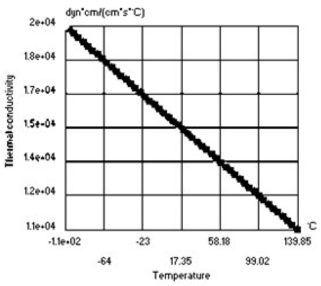  |

**Table S2.** Difference mesh refinement of the automating generated mesh in seven levels.

| Mesh refinement | | | |
|---|---|---|---|
| Level 1 | Level 2 | Level 3 | Level 4 |
| 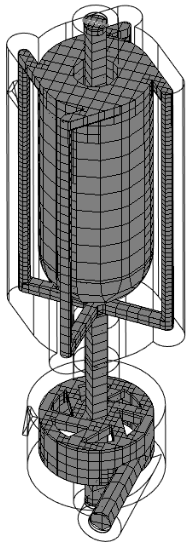 | 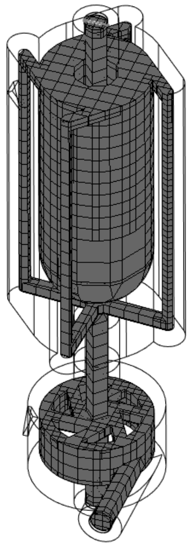 | 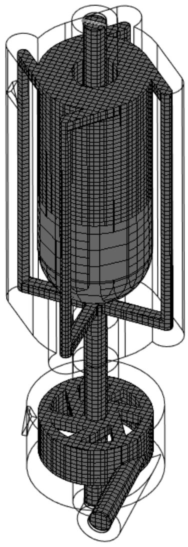 | 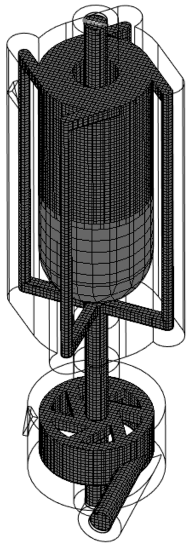 |
| Level 5 | Level 6 | Level 7 | |
| 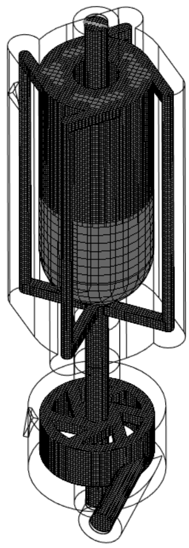 | 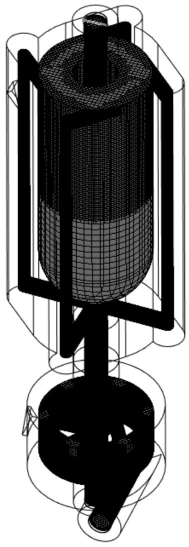 | 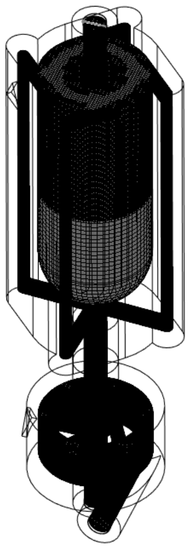 | |

***Table S3.*** Mesh independency test using the vortex tube reactor model.

| **Model** | **Mesh level** | **Mesh cell count** | **Iteration** | **Mass fraction of water** | **Reynold number** | **Convergence plot of mesh independency test using the vortex tube reactor model** |
|---|---|---|---|---|---|---|
| 1 | 1 | 3,268 | 160 | 0.5495 | 1,117.56 | 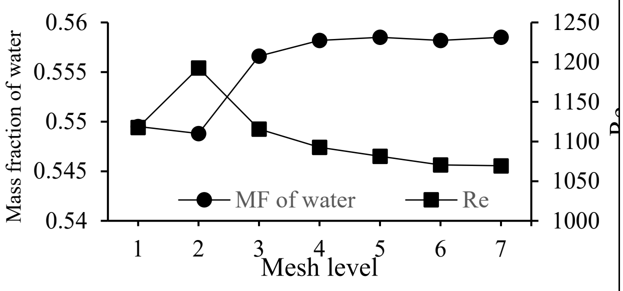 |
| | 2 | 3,558 | 160 | 0.5488 | 1,192.62 | |
| | 3 | 24,010 | 268 | 0.5566 | 1,115.46 | |
| | 4 | 48,429 | 300 | 0.5582 | 1,092.6 | |
| | 5 | 122,228 | 351 | 0.5585 | 1,081.32 | |
| | 6 | 817,569 | 521 | 0.5582 | 1,070.28 | |
| | 7 | 2,236,753 | 695 | 0.5585 | 1,069.44 | |
| 2 | 1 | 3,117 | 127 | 0.5542 | 1,751.05 | 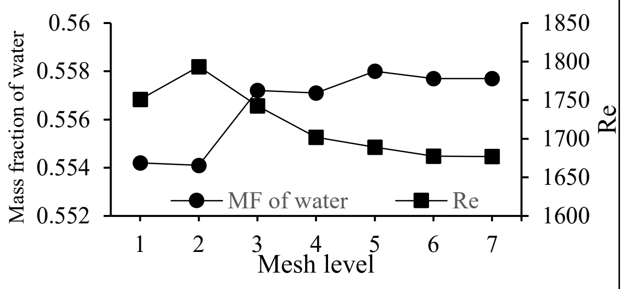 |
| | 2 | 2,677 | 141 | 0.5541 | 1,793.60 | |
| | 3 | 17,398 | 216 | 0.5572 | 1,742.80 | |
| | 4 | 44,850 | 261 | 0.5571 | 1,701.90 | |
| | 5 | 112,741 | 320 | 0.558 | 1,689.10 | |
| | 6 | 821,352 | 508 | 0.5577 | 1,677.65 | |
| | 7 | 2,276,391 | 676 | 0.5577 | 1,676.95 | |
| 3 | 1 | 3,163 | 160 | 0.5537 | 1,213.29 | 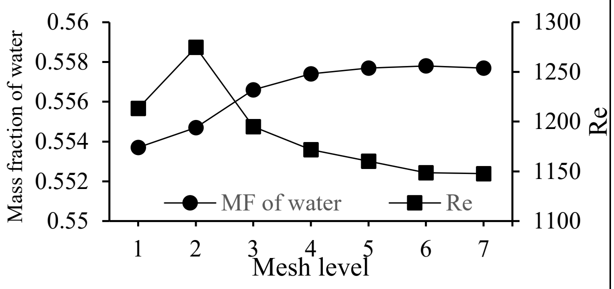 |
| | 2 | 3,320 | 160 | 0.5547 | 1,274.74 | |
| | 3 | 15,409 | 239 | 0.5566 | 1,195.06 | |
| | 4 | 30,693 | 246 | 0.5574 | 1,171.68 | |
| | 5 | 76,805 | 283 | 0.5577 | 1,160.21 | |
| | 6 | 658,343 | 447 | 0.5578 | 1,148.58 | |
| | 7 | 1,828,178 | 620 | 0.5577 | 1,147.72 | |
| 4 | 1 | 3,117 | 126 | 0.5539 | 1,750.41 | 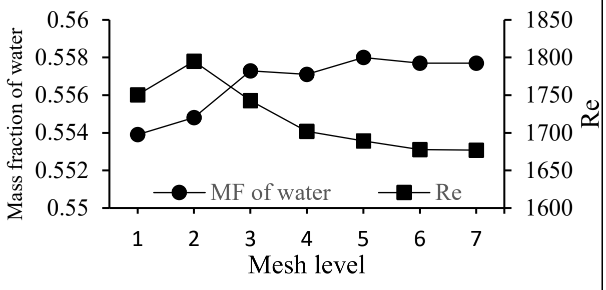 |
| | 2 | 2,677 | 146 | 0.5548 | 1,795.03 | |
| | 3 | 17,377 | 217 | 0.5573 | 1,742.72 | |
| | 4 | 44,878 | 262 | 0.5571 | 1,701.94 | |
| | 5 | 112,391 | 319 | 0.558 | 1,689.10 | |
| | 6 | 821,325 | 508 | 0.5577 | 1,677.65 | |
| | 7 | 2,276,856 | 676 | 0.5577 | 1,676.95 | |
| 5 | 1 | 2,335 | 160 | 0.5559 | 1,222.86 | 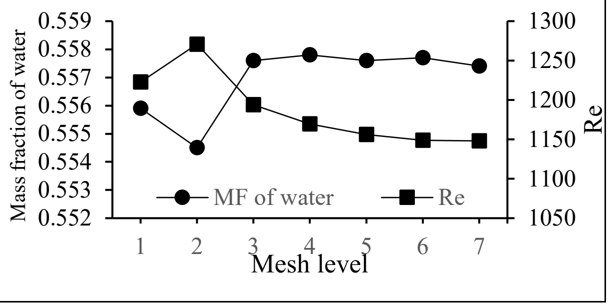 |
| | 2 | 2,435 | 148 | 0.5545 | 1,270.80 | |
| | 3 | 14,856 | 195 | 0.5576 | 1,194.00 | |
| | 4 | 34,843 | 222 | 0.5578 | 1,169.70 | |
| | 5 | 90,903 | 261 | 0.5576 | 1,156.02 | |
| | 6 | 133,316 | 437 | 0.5577 | 1,148.88 | |
| | 7 | 1,997,191 | 598 | 0.5574 | 1,148.04 | |

| Model | Mesh level | Mesh cell count | Iteration | Mass fraction of water | Reynold number | Convergence plot of mesh independency test using the vortex tube reactor model |
|---|---|---|---|---|---|---|
| 6 | 1 | 1,409 | 58 | 0.4574 | 755.68 | 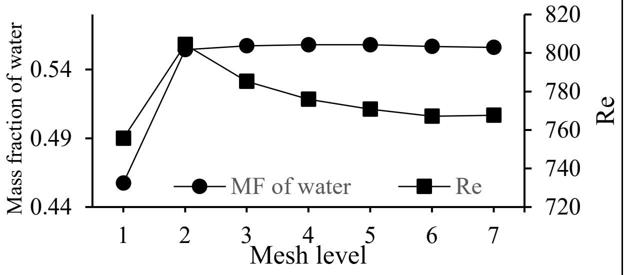 |
| | 2 | 2,418 | 116 | 0.5543 | 804.36 | |
| | 3 | 11,806 | 184 | 0.5571 | 785.24 | |
| | 4 | 32,957 | 201 | 0.558 | 775.96 | |
| | 5 | 85,334 | 226 | 0.558 | 770.76 | |
| | 6 | 849,119 | 383 | 0.5568 | 767.20 | |
| | 7 | 603,021 | 363 | 0.556 | 767.72 | |
| 7 | 1 | 5,039 | 145 | 0.5548 | 1,974.36 | 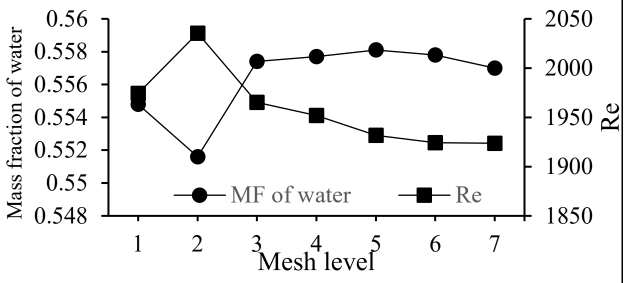 |
| | 2 | 3,664 | 144 | 0.5516 | 2,035.48 | |
| | 3 | 16,898 | 228 | 0.5574 | 1,965.32 | |
| | 4 | 36,375 | 260 | 0.5577 | 1,952.00 | |
| | 5 | 102,972 | 310 | 0.5581 | 1,931.88 | |
| | 6 | 1,031,558 | 488 | 0.5578 | 1,924.36 | |
| | 7 | 2,843,638 | 651 | 0.557 | 1,923.72 | |
| 8 | 1 | 5,498 | 167 | 0.5559 | 792.80 | 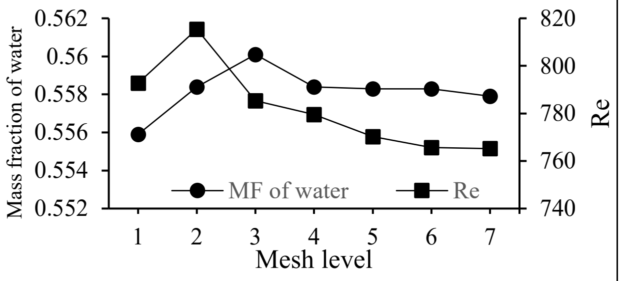 |
| | 2 | 3,687 | 160 | 0.5584 | 815.44 | |
| | 3 | 23,934 | 276 | 0.5601 | 785.36 | |
| | 4 | 53,093 | 292 | 0.5584 | 779.52 | |
| | 5 | 156,980 | 301 | 0.5583 | 770.20 | |
| | 6 | 1,428,243 | 526 | 0.5583 | 765.72 | |
| | 7 | 3,882,523 | 696 | 0.5579 | 765.20 | |
| 9 | 1 | 1,425 | 134 | 0.5581 | 834.60 | 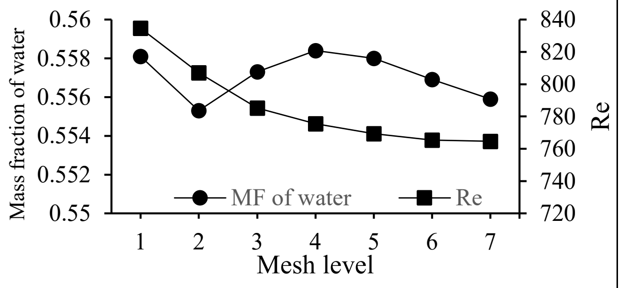 |
| | 2 | 2,591 | 123 | 0.5553 | 807.08 | |
| | 3 | 15,666 | 200 | 0.5573 | 785.24 | |
| | 4 | 45,878 | 220 | 0.5584 | 775.44 | |
| | 5 | 120,732 | 248 | 0.558 | 769.36 | |
| | 6 | 1,010,688 | 419 | 0.5569 | 765.36 | |
| | 7 | 884,499 | 414 | 0.5559 | 764.64 | |
| 10 | 1 | 3,117 | 136 | 0.5558 | 3,270.55 | |
| | 2 | 2,677 | 160 | 0.5562 | 3,345.20 | |
| | 3 | 17,377 | 244 | 0.558 | 3,246.45 | |
| | 4 | 44,822 | 328 | 0.5581 | 3,168.95 | |
| | 5 | 113,014 | 389 | 0.5584 | 3,148.10 | |
| | 6 | 821,352 | 534 | 0.5586 | 3,132.25 | |

| | | | | | | |
|---|---|---|---|---|---|---|
| | 7 | 2,276,551 | 823 | 0.5581 | 3,139.20 | 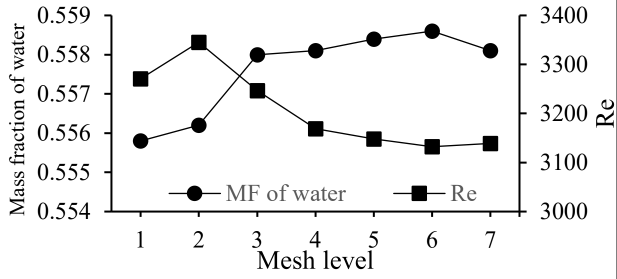 |

| Mode l | Mesh level | Mesh cell count | Iteration | Mass fraction of water | Reynold number | Convergence plot of mesh independency test using the vortex tube reactor model |
|---|---|---|---|---|---|---|
| 11 | 1 | 2,275 | 160 | 0.5556 | 3,033.30 | 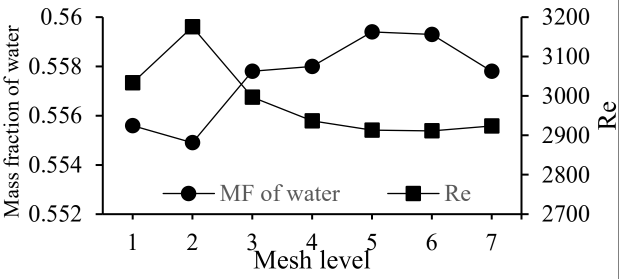 |
| | 2 | 2,187 | 159 | 0.5549 | 3,175.20 | |
| | 3 | 9,708 | 191 | 0.5578 | 2,997.30 | |
| | 4 | 22,226 | 223 | 0.558 | 2,936.70 | |
| | 5 | 59,830 | 311 | 0.5594 | 2,912.88 | |
| | 6 | 639,576 | 461 | 0.5593 | 2,911.32 | |
| | 7 | 1,747,745 | 571 | 0.5578 | 2,923.44 | |
| 12 | 1 | 3,163 | 160 | 0.5516 | 3,018.54 | 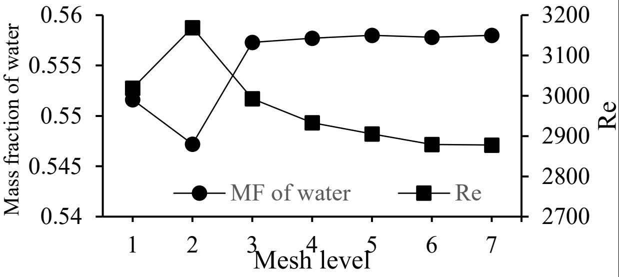 |
| | 2 | 3,230 | 160 | 0.5472 | 3,169.38 | |
| | 3 | 15,416 | 239 | 0.5573 | 2,992.98 | |
| | 4 | 30,406 | 293 | 0.5577 | 2,932.80 | |
| | 5 | 76,609 | 361 | 0.558 | 2,905.38 | |
| | 6 | 658,350 | 531 | 0.5578 | 2,879.04 | |
| | 7 | 1,828,150 | 693 | 0.558 | 2,877.78 | |
| 13 | 1 | 1,409 | 130 | 0.5543 | 2,090.64 | 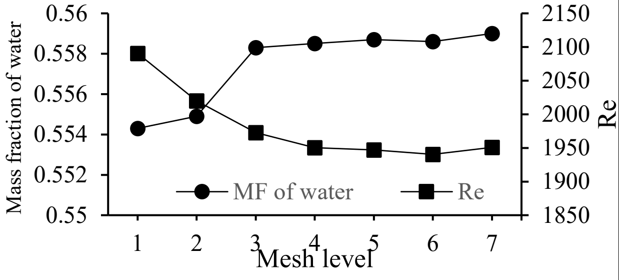 |
| | 2 | 2,418 | 118 | 0.5549 | 2,020.12 | |
| | 3 | 11,806 | 201 | 0.5583 | 1,972.92 | |
| | 4 | 32,957 | 230 | 0.5585 | 1,950.56 | |
| | 5 | 85,334 | 279 | 0.5587 | 1,947.00 | |
| | 6 | 849,077 | 434 | 0.5586 | 1,940.40 | |
| | 7 | 602,989 | 474 | 0.559 | 1,950.80 | |
| 14 | 1 | 4,928 | 163 | 0.5564 | 795.28 | |
| | 2 | 3,664 | 147 | 0.5552 | 819.28 | |
| | 3 | 16,898 | 254 | 0.5579 | 786.20 | |
| | 4 | 36,361 | 258 | 0.5581 | 780.48 | |
| | 5 | 102,953 | 268 | 0.5583 | 770.92 | |

| | | | | | | |
|---|---|---|---|---|---|---|
| | 6 | 1,032,001 | 459 | 0.5582 | 766.20 | 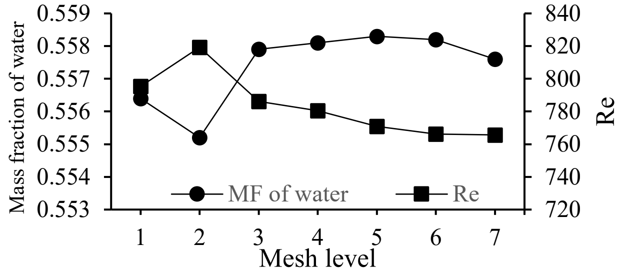 |
| | 7 | 2,844,953 | 624 | 0.5576 | 765.68 | |
| 15 | 1 | 3,117 | 140 | 0.555 | 252.40 | 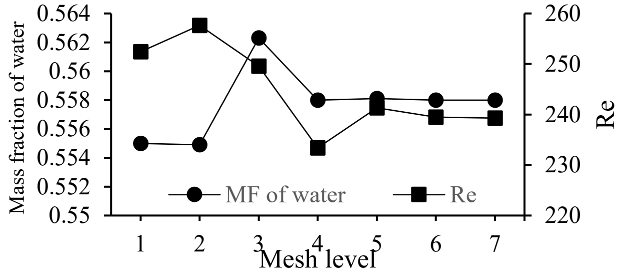 |
| | 2 | 2,677 | 136 | 0.5549 | 257.65 | |
| | 3 | 17,377 | 244 | 0.5623 | 249.60 | |
| | 4 | 44,822 | 202 | 0.558 | 233.40 | |
| | 5 | 112,839 | 239 | 0.5581 | 241.35 | |
| | 6 | 821,352 | 401 | 0.558 | 239.45 | |
| | 7 | 2,277,101 | 536 | 0.558 | 239.30 | |

| **Model** | **Mesh level** | **Mesh cell count** | **Iteration** | **Mass fraction of water** | **Reynold number** | **Convergence plot of mesh independency test using the vortex tube reactor model** |
|---|---|---|---|---|---|---|
| 16 | 1 | 1,271 | 130 | 0.5555 | 2,084.84 | 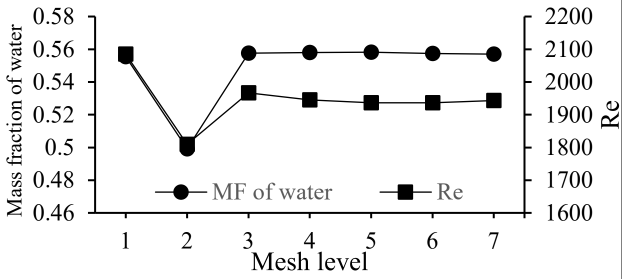 |
| | 2 | 2,503 | 41 | 0.4992 | 1,809.56 | |
| | 3 | 15,572 | 195 | 0.5576 | 1,967.12 | |
| | 4 | 45,731 | 232 | 0.558 | 1,945.00 | |
| | 5 | 121,093 | 303 | 0.5582 | 1,936.92 | |
| | 6 | 1,010,660 | 449 | 0.5574 | 1,936.08 | |
| | 7 | 866,158 | 441 | 0.5571 | 1,942.88 | |
| 17 | 1 | 3,117 | 134 | 0.555 | 1,760.80 | 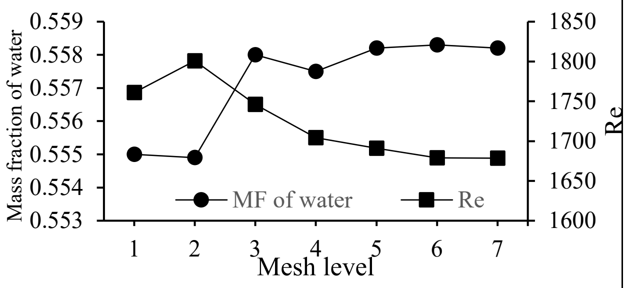 |
| | 2 | 2,677 | 146 | 0.5549 | 1,800.80 | |
| | 3 | 17,398 | 244 | 0.558 | 1,746.10 | |
| | 4 | 44,815 | 273 | 0.5575 | 1,704.20 | |
| | 5 | 112,965 | 339 | 0.5582 | 1,690.90 | |
| | 6 | 821,352 | 506 | 0.5583 | 1,679.05 | |
| | 7 | 2,276,793 | 681 | 0.5582 | 1,678.55 | |
| 18 | 1 | 927 | 134 | 0.555 | 2,059.35 | |
| | 2 | 1,654 | 102 | 0.5557 | 1,763.00 | |
| | 3 | 10,552 | 166 | 0.5584 | 1,733.30 | |
| | 4 | 29,829 | 188 | 0.5585 | 1,721.45 | |

| | | | | | | |
|---|---|---|---|---|---|---|
| | 5 | 72,492 | 217 | 0.5586 | 1,730.95 | 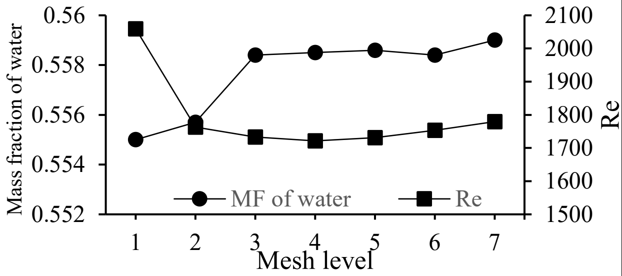  |
| | 6 | 195,603 | 275 | 0.5584 | 1,753.35 | |
| | 7 | 533,735 | 373 | 0.559 | 1,779.45 | |
| 19 | 1 | 4,420 | 163 | 0.5045 | 1,745.15 | 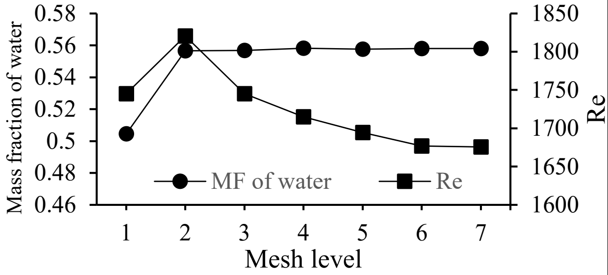  |
| | 2 | 3,757 | 160 | 0.5567 | 1,820.60 | |
| | 3 | 23,927 | 267 | 0.5569 | 1,745.05 | |
| | 4 | 57,463 | 354 | 0.5582 | 1,715.00 | |
| | 5 | 150,026 | 404 | 0.5576 | 1,694.40 | |
| | 6 | 941,032 | 584 | 0.5581 | 1,676.70 | |
| | 7 | 2,541,242 | 814 | 0.5581 | 1,675.65 | |
| 20 | 1 | 3,047 | 133 | 0.5548 | 1,762.20 | 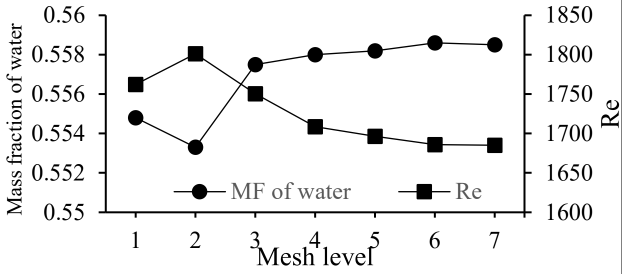  |
| | 2 | 2,389 | 130 | 0.5533 | 1,801.15 | |
| | 3 | 10,147 | 200 | 0.5575 | 1,750.60 | |
| | 4 | 22,750 | 247 | 0.558 | 1,708.75 | |
| | 5 | 60,695 | 268 | 0.5582 | 1,696.50 | |
| | 6 | 673,532 | 437 | 0.5586 | 1,685.70 | |
| | 7 | 1,882,891 | 552 | 0.5585 | 1,685.10 | |

| **Model** | **Mesh level** | **Mesh cell count** | **Iteration** | **Mass fraction of water** | **Reynold number** | **Convergence plot of mesh independency test using the vortex tube reactor model** |
|---|---|---|---|---|---|---|
| 21 | 1 | 3,117 | 131 | 0.5547 | 1,760.8 | 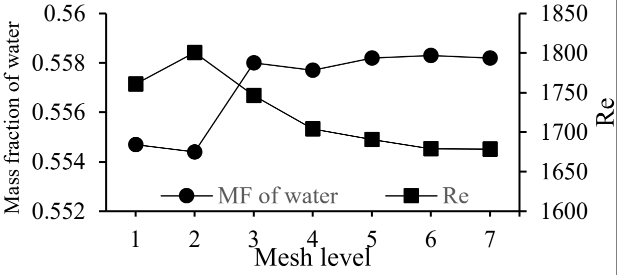  |
| | 2 | 2,677 | 143 | 0.5544 | 1,800.65 | |
| | 3 | 17,398 | 244 | 0.558 | 1,746.10 | |
| | 4 | 44,822 | 280 | 0.5577 | 1,704.25 | |
| | 5 | 112,741 | 342 | 0.5582 | 1,690.90 | |
| | 6 | 821,366 | 506 | 0.5583 | 1,679.00 | |
| | 7 | 2,277,094 | 680 | 0.5582 | 1,678.55 | |
| 22 | 1 | 3,258 | 160 | 0.5453 | 3,042.18 | |
| | 2 | 3,558 | 160 | 0.5423 | 3,206.94 | |
| | 3 | 23,821 | 267 | 0.5575 | 2,997.66 | |
| | 4 | 48,992 | 335 | 0.5579 | 2,934.78 | |

| | | | | | | |
|---|---|---|---|---|---|---|
| | 5 | 122,312 | 452 | 0.5583 | 2,905.02 | 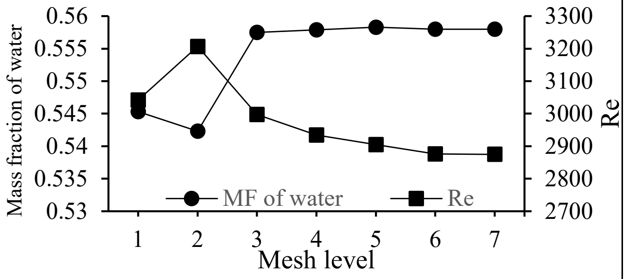 |
| | 6 | 817,667 | 636 | 0.558 | 2,876.46 | |
| | 7 | 2,236,669 | 867 | 0.558 | 2,874.78 | |
| 23 | 1 | 2,335 | 160 | 0.5549 | 3,044.70 | 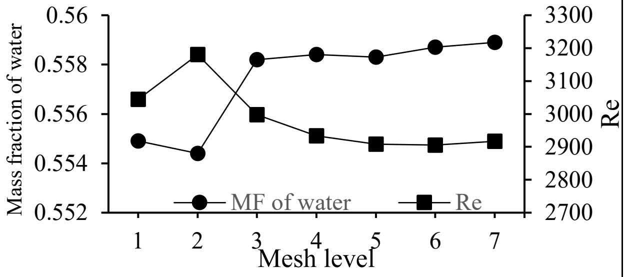 |
| | 2 | 2,435 | 160 | 0.5544 | 3,180.12 | |
| | 3 | 14,835 | 230 | 0.5582 | 2,997.96 | |
| | 4 | 34,836 | 270 | 0.5584 | 2,933.40 | |
| | 5 | 90,896 | 331 | 0.5583 | 2,908.20 | |
| | 6 | 733,085 | 518 | 0.5587 | 2,905.50 | |
| | 7 | 1,997,415 | 670 | 0.5589 | 2,916.90 | |
| 24 | 1 | 1,446 | 135 | 0.5559 | 1,110.75 | 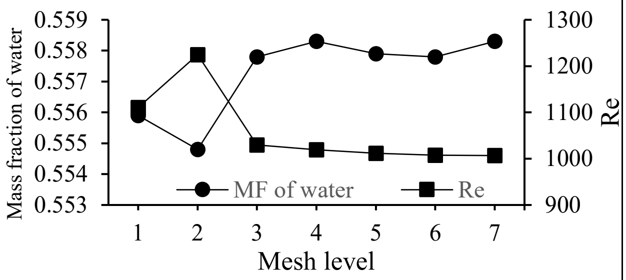 |
| | 2 | 3,138 | 114 | 0.5548 | 1,224.63 | |
| | 3 | 15,544 | 210 | 0.5578 | 1,029.81 | |
| | 4 | 35,977 | 236 | 0.5583 | 1,019.04 | |
| | 5 | 101,130 | 262 | 0.5579 | 1,011.36 | |
| | 6 | 276,519 | 307 | 0.5578 | 1,007.88 | |
| | 7 | 750,381 | 395 | 0.5583 | 1,006.74 | |
| 25 | 1 | 2,275 | 158 | 0.5557 | 1,223.34 | 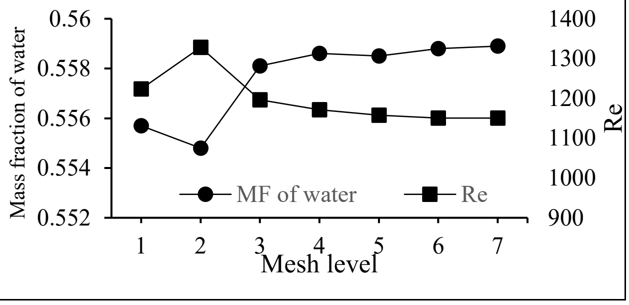 |
| | 2 | 2,187 | 147 | 0.5548 | 1,328.04 | |
| | 3 | 9,729 | 201 | 0.5581 | 1,196.64 | |
| | 4 | 22,184 | 209 | 0.5586 | 1,171.02 | |
| | 5 | 59,858 | 237 | 0.5585 | 1,157.82 | |
| | 6 | 639,597 | 381 | 0.5588 | 1,150.86 | |
| | 7 | 1,747,738 | 520 | 0.5589 | 1,150.50 | |

| Model | Mesh level | Mesh cell count | Iteration | Mass fraction of water | Reynold number | Convergence plot of mesh independency test using the vortex tube reactor model |
|---|---|---|---|---|---|---|
| 26 | 1 | 5,387 | 164 | 0.5544 | 1,984.04 | |
| | 2 | 3,687 | 160 | 0.5527 | 2,045.56 | |
| | 3 | 23,934 | 276 | 0.5574 | 1,965.12 | |
| | 4 | 53,100 | 326 | 0.5583 | 1,951.28 | |

| | | | | | | |
|---|---|---|---|---|---|---|
| | 5 | 156,961 | 366 | 0.5584 | 1,927.64 | 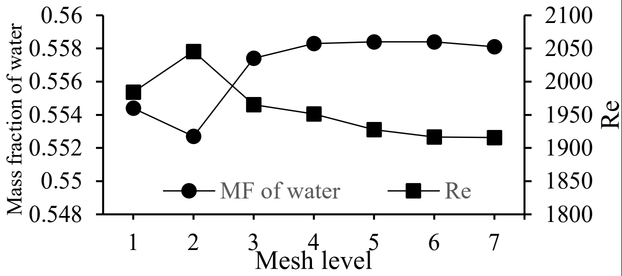 |
| | 6 | 1,427,656 | 580 | 0.5584 | 1,916.36 | |
| | 7 | 3,879,258 | 786 | 0.5581 | 1,915.76 | |
| 27 | 1 | 3,187 | 137 | 0.5558 | 1,761.15 | 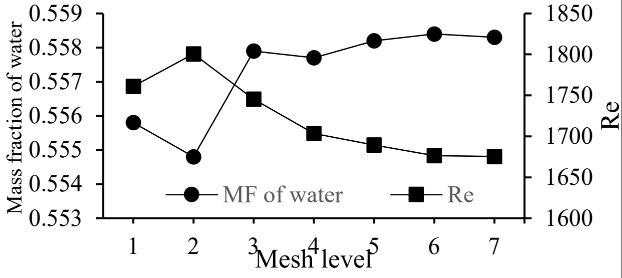 |
| | 2 | 2,890 | 147 | 0.5548 | 1,800.65 | |
| | 3 | 24,677 | 268 | 0.5579 | 1,745.20 | |
| | 4 | 66,594 | 312 | 0.5577 | 1,703.315 | |
| | 5 | 170,598 | 378 | 0.5582 | 1,689.45 | |
| | 6 | 1,013,285 | 557 | 0.5584 | 1,676.45 | |
| | 7 | 2,784,134 | 765 | 0.5583 | 1,675.35 | |
| 28 | 1 | 2,603 | 160 | 0.554 | 2,528.96 | 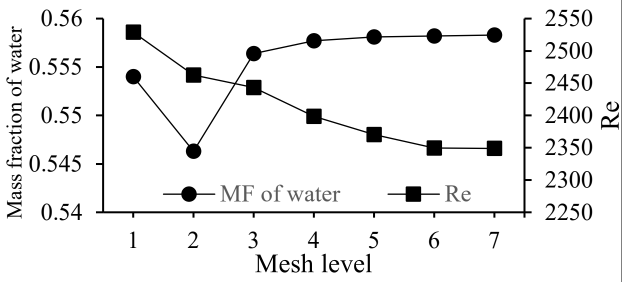 |
| | 2 | 2,263 | 160 | 0.5463 | 2,462.39 | |
| | 3 | 12,410 | 225 | 0.5564 | 2,443.49 | |
| | 4 | 27,077 | 297 | 0.5577 | 2,398.55 | |
| | 5 | 70,760 | 393 | 0.5581 | 2,370.55 | |
| | 6 | 630,716 | 624 | 0.5582 | 2,349.69 | |
| | 7 | 1,673,348 | 817 | 0.5583 | 2,348.85 | |
| 29 | 1 | 3,117 | 129 | 0.5542 | 1,759.85 | 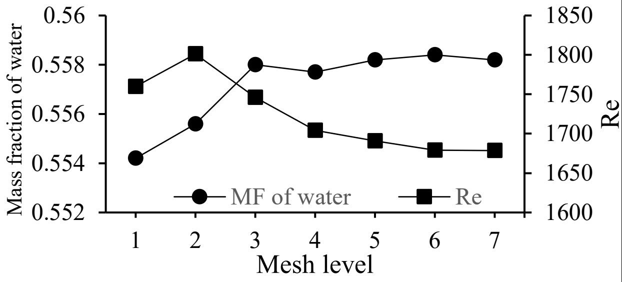 |
| | 2 | 2,677 | 153 | 0.5556 | 1,801.15 | |
| | 3 | 17,377 | 244 | 0.558 | 1,746.10 | |
| | 4 | 44,850 | 274 | 0.5577 | 1,704.25 | |
| | 5 | 112,363 | 340 | 0.5582 | 1,690.90 | |
| | 6 | 821,352 | 506 | 0.5584 | 1,679.05 | |
| | 7 | 2,277,087 | 681 | 0.5582 | 1,678.55 | |
| 30 | 1 | 3,117 | 132 | 0.5549 | 1,760.95 | 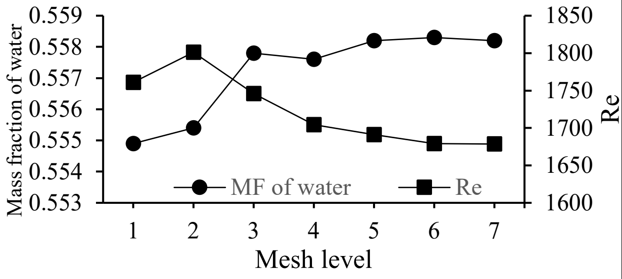 |
| | 2 | 2,677 | 152 | 0.5554 | 1,801.10 | |
| | 3 | 17,377 | 240 | 0.5578 | 1,746.10 | |
| | 4 | 44,850 | 274 | 0.5576 | 1,704.20 | |
| | 5 | 112,650 | 343 | 0.5582 | 1,690.90 | |
| | 6 | 821,086 | 506 | 0.5583 | 1,679.00 | |
| | 7 | 2,276,912 | 681 | 0.5582 | 1,678.55 | |
